# Hybrid Nanofluid Thermal Conductivity and Optimization: Original Approach and Background

**DOI:** 10.3390/nano12162847

**Published:** 2022-08-18

**Authors:** Jake Wohld, Joshua Beck, Kallie Inman, Michael Palmer, Marcus Cummings, Ryan Fulmer, Saeid Vafaei

**Affiliations:** Mechanical Engineering Department, Bradley University, Peoria, IL 61606, USA

**Keywords:** nanofluid, hybrid nanofluid, thermal conductivity, viscosity, effect of nanofluids, optimization, theoretical predictions, models, nanoparticles

## Abstract

The focus of this paper was to develop a comprehensive nanofluid thermal conductivity model that can be applied to nanofluids with any number of distinct nanoparticles for a given base fluid, concentration, temperature, particle material, and particle diameter. For the first time, this model permits a direct analytical comparison between nanofluids with a different number of distinct nanoparticles. It was observed that the model’s average error was ~5.289% when compared with independent experimental data for hybrid nanofluids, which is lower than the average error of the best preexisting hybrid nanofluid model. Additionally, the effects of the operating temperature and nanoparticle concentration on the thermal conductivity and viscosity of nanofluids were investigated theoretically and experimentally. It was found that optimization of the operating conditions and characteristics of nanofluids is crucial to maximize the heat transfer coefficient in nanofluidics and microfluidics. Furthermore, the existing theoretical models to predict nanofluid thermal conductivity were discussed based on the main mechanisms of energy transfer, including Effective Medium Theory, Brownian motion, the nanolayer, aggregation, Molecular Dynamics simulations, and enhancement in hybrid nanofluids. The advantage and disadvantage of each model, as well as the level of accuracy of each model, were examined using independent experimental data.

## 1. Introduction

Fluids are used in many thermal management systems to control and enhance the rate of heat transfer. In modern systems, recent technological developments have increased the amount of heat output typically seen [1]. Hence, as the amount of power dissipation per unit size increases, higher rates of heat transfer are necessary to prevent overheating. As a result, the demand for fluids with high heat transfer is continuing to increase, and it is becoming more difficult for standard working fluids to fulfill these needs. It was discovered in 1995 by Choi and Eastman [2] that adding solid nanoparticles (typically less than 100 nm in size) to a fluid increases its effective thermal conductivity. This new class of fluids commonly goes by the name ”nanofluids”. As such, nanofluids are becoming more prominent for their potential use as working fluids [3]. Their capacity for higher thermal conductivity allows them to better meet modern thermal management needs [2]. Although, while nanofluids do have higher thermal conductivity, they also generally have a higher viscosity [4]. Therefore, their ability to transfer heat efficiently declines, since there is less energy transfer between fluid layers. So, when considering the use of nanofluids in heat transfer applications, it is critical to examine both the thermal conductivity and the viscosity in typical working conditions. In order to obtain the best results out of the nanofluid used, the effects on each characteristic must be analyzed and optimized against each other. Thus, in this paper, a review on the optimization of nanofluids is carried out, and a recommendation is made on how to best engineer a nanofluid. In addition, thermal properties [5,6] and viscosity [7] also play a significant role in different phenomena.

Again, nanofluids are most commonly consist of two parts. There are the nanoparticles and the base liquid they are suspended in. Occasionally, a surfactant is also used to keep the nanoparticles from clumping [8]. The nanoparticles used in nanofluids generally have significantly higher thermal conductivities than that of the base fluids. Common materials are metals or metal oxides, as originally experimented with by Choi and Eastman [2]. Additionally, advanced carbon-based materials such as nanodiamonds, graphene, and carbon nanotubes are taken advantage of [9,10,11]. The base fluids used in nanofluids are generally similar to working fluids that have been previously applied in thermal management systems, including water, ethylene glycol, and oils [12,13]. Although these base fluids struggle to meet modern heat transfer demands, the addition of nanoparticles enhances the fluids’ thermal conductivity to allow them to better meet those needs.

There are various types of models that predict the nanofluid thermal conductivity. Specifically, the models consider: Effective Medium Theory (EMT) [14,15,16,17,18], Brownian motion [19,20,21,22,23,24] the nanolayer [25,26,27,28], the aggregation of nanoparticles [29,30,31,32], molecular dynamics simulations [33,34,35,36], and hybrid nanofluids [37,38,39]. EMT consists of of averaging the properties of a composite material’s constituents, with the goal of obtaining sufficiently accurate estimates of the macroscopic properties [40]. Brownian motion models consider how the random motion of particles alters the energy transfer within a stationary fluid, and hence the thermal conductivity [41]. Nanolayer models account for the effects of an ordered layer of base liquid molecules that forms on the surface of the nanoparticles [42]. It has been proposed that this thin layer could effectively enhance thermal conductivity [25]. Aggregation models consider the clustering of nanoparticles, which occurs due to particle transportation and inter-particle forces [43]. However, there exists a disagreement on when aggregation increases or decreases the thermal conductivity [30,44]. With the intent of modeling many of these mechanisms simultaneously, researchers use Molecular Dynamics Simulations to simulate every particle interaction in a nanofluid system. This is performed by solving the classical equations of motion at the atomic scale [45]. Additionally, researchers have begun theoretically investigating the hybrid nanofluid thermal conductivity, as hybrid nanofluids generally perform better than mono nanofluids [37,46,47]. Finally, theoretical investigations have also been carried out on the viscosity of nanofluids [48,49,50,51,52].

To accurately describe the seemingly anomalous thermal conductivity enhancement, researchers develop nanofluid thermal conductivity models by taking advantage of a wide range of techniques. For instance, many models are derived from heat transfer networks considering one or multiple enhancement mechanisms. In other cases, statistical analysis is performed on large sets of data to arrive at an empirical model. Occasionally, these two techniques are combined, as some of the thermal network characteristics are difficult to measure with current methods. In addition, computational simulations are completed with the aim of modeling the interactions between each particle in a nanofluid system. While these techniques are covered in detail in this paper, various artificial intelligence models have been recently explored by researchers to predict the nanofluid thermal conductivity [53,54,55].

There are several dominating theories regarding the primary mechanism through which nanoparticles enhance the nanofluid thermal conductivity. One theory is based on the electrical conductivity of a mixed medium, EMT as pioneered by Maxwell [14]. It suggests that thermal conductivity enhancement is primarily caused by high thermal conductivity particles increasing the thermal diffusion of the nanofluid. Some researchers expanded the results of this theory, such as Hamilton and Crosser [15], who included the effects of particle shape. Another theory, described by Jang and Choi [41], proposes that the Brownian motion of nanoparticles in a fluid can lead to microconvection, therefore increasing the thermal conductivity. There are many ways this mechanism is incorporated into theoretical models. Koo and Kleinstreuer [21], for instance, considered the enhancement due to Brownian motion to be additive to that predicted by EMT. Furthermore, Leong et al. [25] thought that the tendency of the base fluid to form a dense nanolayer around the nanoparticles may lead to thermal conductivity enhancement. This is because the molecular structure of the nanolayer is highly ordered. As a result, the thermal conductivity of the nanolayer would be increased compared to the base fluid thermal conductivity. Yu and Choi [27] considered the nanolayer by replacing the actual nanoparticles with equivalent nanoparticles. These equivalent nanoparticles have properties resulting from the combination of the nanoparticle itself, and the nanolayer covering the nanoparticle. The aggregation of nanoparticles is another thermal conductivity-altering mechanism widely considered. One theory suggests heat transfer occurs efficiently through the aggregates due to the lower resistance path within, as described by Keblinski et al. [44]. However, particle aggregation has been found to reduce Brownian motion, potentially leading to a decrease in thermal conductivity [30]. Prasher et al. [29] believed the benefits due to aggregation outweighed the negatives when formulating their model. They also chose to model aggregation as a time-dependent process. However, not all researchers use this approach, as seen with the other aggregation models described in this paper. While all these mechanisms are thought to improve the nanofluid thermal conductivity relative to the base fluid in some form, it remains undetermined which mechanism has the most dominant effect. To take into account all of these mechanisms, researchers are beginning to simulate the molecular dynamics of nanoparticle/base fluid systems. Lin et al. [35] used this method, and they were able to simultaneously model the thermal conductivity enhancement due to EMT and the nanolayer. Furthermore, as nanofluid research continues to progress and thermal management demands get even higher, the effects of using multiple nanoparticle types in one nanofluid are being explored. These fluids are commonly deemed ”hybrid nanofluids”. The mechanisms behind thermal conductivity enhancement in a mono nanofluid still apply to hybrid nanofluids. However, there is an additional effect due to the different particle types interacting with each other. In these cases, the particles may enhance the properties of the nanofluid more than what would be observed if their standalone effects were combined. This is commonly termed as the synergistic effect [56]. Because of the significantly high number of characteristics required to describe hybrid nanofluids, many hybrid nanofluid thermal conductivity models tend to focus on specific combinations of particles and conditions. In this paper, a general hybrid nanofluid model is developed to address this issue. It can predict the thermal conductivity for a variety of environmental conditions and particle combinations, including use in ternary hybrid nanofluids and nanofluids with N distinct nanoparticles. It also maintains a higher level of accuracy than the existing general hybrid nanofluid models over a significantly broader validity range.

Similarly to thermal conductivity enhancement, there are several mechanisms by which the introduction of nanoparticles is thought to influence the viscosity of nanofluids. Some of the models investigated consider the Brownian motion of the nanoparticles, as well as the nanolayer and EMT. However, other commonly considered mechanisms include kinetic gas theory, local composition, and binary mixing [57]. The effects of nanoparticles on nanofluid viscosity are somewhat disagreed upon depending on the characteristic investigated. However, Mishra et al. [58] found that an increase in nanofluid viscosity with an increasing particle concentration is widely reported in the literature. An even stronger agreement was observed with the effects of temperature. It is clear from many existing studies that an increase in temperature decreases the viscosity of the nanofluid [58]. Brinkman [48] created an early nanofluid viscosity model, where it was suggested that the nanofluid viscosity is solely dependent on the nanoparticle volume concentration and the base fluid viscosity. Meybodi et al. [49] proposed a more complex model, additionally considering the effects of concentration and temperature. They also accounted for particle size, where it was predicted that as the nanoparticle diameter increases, the nanofluid viscosity increases. Masoumi et al. [50] developed their model to consider the effects of Brownian motion on the nanofluid viscosity. Nanoparticle size and density, volume fraction, and temperature were all accounted for. A correction factor, found by analyzing experimental data was also taken advantage of. Hosseini et al. [51] proposed an empirical model based on dimensionless groups considering the temperature, nanoparticle size, volume fraction, base liquid viscosity, and the nanolayer. Yet another model was created by Klazly and Bognár [52] that considered nanofluid viscosity to be a function of nanoparticle size and material, base fluid properties, temperature, particle density, and the nanolayer thickness.

In summary, many theoretical models have been proposed for both the thermal conductivity [14,19,25,32,33] and the viscosity [48,49,50,51,52] of nanofluids. Although some of these models only consider one of the mechanisms described, many take multiple mechanisms into consideration to improve their accuracy as experimental conditions change [21,29,30,31]. Some of the models examined were created by beginning with known laws of heat transfer and fluid dynamics, but most utilized gathered experimental data and used regression analysis to further formulate the models [20,23,24,36]. In this paper, models for the thermal conductivity and viscosity of nanofluids as well as models for the thermal conductivity of hybrid nanofluids were reviewed. Additionally, their levels of accuracy were measured and compared using existing, independent experimental data. Comparisons between models were performed using the percent mean average error (MAE) for several datasets in each model category. Ultimately, the trends observed in the models and the experimental datasets were used to investigate methods of optimizing the effects of nanoparticles on the thermal conductivity and viscosity of nanofluids.

## 2. Nanofluid Thermal Conductivity Models

In this section, thermal conductivity models are categorized into six groups namely, Effective Medium Theory, Brownian models, nanolayer models, aggregation models, models resulting from Molecular Dynamics simulations, and hybrid nanofluid models. A brief discussion on the background of each model is provided before they are compared to existing experimental data from independent sources. The level of accuracy is measured using percent mean average error (MAE), and is represented by the following equation:

(1) $$\mathrm{MAE}=\frac{100}{X}\overset{X}{\underset{i=1}{\sum }}\left|\frac{k_{\mathrm{th},i}-k_{\exp,i}}{k_{\exp,i}}\right|,$$

where X is the number of data points, $k_{\mathrm{th},i}$ is the value predicted by theory, and $k_{\exp,i}$ is the experimentally observed value. Details on nomenclature conventions can be seen in Appendix A, Table A1. Meanwhile, a brief overview on each thermal conductivity model can be seen in Appendix B, Table A2.

### 2.1. Effective Medium Theory

The earliest attempts to predict the thermal conductivities of nanofluids were made using Effective Medium Theory (EMT). EMT models the nanofluid thermal conductivity by trying to predict the effects of the concentration of nanoparticles present on the thermal conductivity compared to the original base fluid. EMT was developed by Maxwell [14] in his 1873 book *A Treatise on Electricity and Magnetism* [14]. His model was originally based on the electrical conductivity of a two-part mixture, but was also found to be analogous to the thermal conductivity of two-component mixtures. To create his model, Maxwell [14] assumed that the current between two elements of a mixture in contact with each other must be continuous. He then solved the continuity equation based on this assumption. To solve the continuity equation, Maxwell [14] assumed that the particles only interacted with the base fluid and not with each other, an assumption which is valid only for low volume fractions of particles with nearly homogeneous distributions. The equation was also solved assuming the particle shape was roughly spherical. The particles used at the time had diameters of millimeters to micrometers. However, particles of this size tend to have severe clumping issues. Instead, nanoparticles are currently used as an alternative, for which the model is still found to be applicable. Therefore, the Maxwell model [14] is given as:

(2) $$k_{nf}=k_{bf}\frac{k_{p}+2k_{bf}+2\phi \left(k_{p}-k_{bf}\right)}{k_{p}+2k_{bf}-\phi \left(k_{p}-k_{bf}\right)},$$

where k denotes the thermal conductivity, with subscripts nf, bf, and p denoting the nanofluid mixture, base fluid, and nanoparticles, respectively, and ϕ denotes the volume fraction of the solid nanoparticles. The advantage of this model is its simplicity. However, it does not account for the particle size or shape, which is a disadvantage. Additionally, the model assumes that the nanoparticles are well separated and non-interacting. Consequently, the model’s accuracy is expected to decrease with increasing concentrations.

Maxwell’s thermal conductivity model provided a basis that other models could expand upon to obtain more accurate predictions. Hamilton and Crosser [15] modified Maxwell’s model by eliminating the assumption of spherical particles. In other words, the effect of particle shape is considered. This effect is included by applying the empirical shape factor n, given as:

(3) $$n=\frac{3}{\psi },$$

(4) $$\psi =\frac{A_{s,sph}}{A_{s,p}},$$

where ψ is the sphericity of the solid particles, defined as the inverse of the ratio of the surface area of the particle to the surface area of a perfect sphere with the same volume. By definition, n can be taken as 3 for spherical particles. Otherwise, it would depend on the specific geometry of other particle shapes. The effects of the particle shape become especially important when the thermal conductivity of the nanoparticle is much higher than that of the base fluid. The Hamilton and Crosser [15] model is given as the following:

(5) $$k_{nf}=k_{bf}\frac{k_{p}+\left(n-1\right)k_{bf}-\left(n-1\right)\left(k_{bf}-k_{p}\right)\phi }{k_{p}+\left(n-1\right)k_{bf}+\left(k_{bf}-k_{p}\right)\phi }.$$

It should be noted that for the case of spherical particles, the Hamilton and Crosser [15] model simplifies to the Maxwell [14] model in Equation (2). The Hamilton and Crosser [15] model predicts that reducing the sphericity of the nanoparticles increases the nanofluid’s thermal conductivity. This is because it is theorized that the surface area to volume ratio is proportional to the thermal conductivity. The primary advantage of this model is that it accounts for the particle shape. However, like the Maxwell [14] model, it assumes the nanoparticles are well separated and non-interacting. Therefore, the accuracy of the Hamilton and Crosser [15] model is also expected to decrease with increasing concentrations.

Similar to Hamilton and Crosser [15], Jeffery [16] based his EMT model on Maxwell’s work. Instead of considering various particle shapes, he sought to improve Maxwell’s model by considering the interactions between the particles themselves. Like Maxwell, Jeffery [16] assumed a static and roughly homogeneous distribution of spherical particles. These particles were assumed to be suspended in a matrix of uniform thermal conductivity representing the base fluid. Their interactions were used to find the heat flux and subsequently the thermal conductivity through the suspension. The model produced using this method was an infinite series. For data analysis, the model was simplified by removing higher-order terms, since they approached zero. The Jeffery [16] model is given as:

(6) $$k_{nf}=k_{bf}\left(1+3\frac{k_{p}-k_{bf}}{k_{p}-2k_{bf}}\phi +\phi^{2}\left(3{\left(\frac{k_{p}-k_{bf}}{k_{p}-2k_{bf}}\right)}^{2}+\frac{3}{4}{\left(\frac{k_{p}-k_{bf}}{k_{p}-2k_{bf}}\right)}^{3}+\frac{9}{16}{\left(\frac{k_{p}-k_{bf}}{k_{p}-2k_{bf}}\right)}^{3}\left(\frac{\frac{k_{p}}{k_{bf}}+2}{2\frac{k_{p}}{k_{bf}}+3}\right)\right)\right).$$

The advantage of this model is that it considers nanoparticle interactions, unlike the previous EMT models [14,15]. Therefore, its accuracy is expected to be higher in comparison for larger volume concentrations.

As discussed earlier, the Maxwell [14] model made large assumptions regarding particle volume fraction, mixture homogeneity, and particle shape. However, it still took the exact particle conductivity as a parameter. Timofeeva et al. [17] decided to make the additional assumption that the particle’s thermal conductivity could be approximated as infinite compared to the base fluid’s thermal conductivity. This assumption was based on an estimation that the change in the thermal conductivity due to particle motion was negligible. This estimation was made after investigating an Al_2_O_3_–water nanofluid mixture. This contrasts with the previous EMT models, which reduced the number of assumptions made by Maxwell [14]. Based on the assumptions, the Timofeeva et al. [17] model is given as:

(7) $$k_{nf}=k_{bf}\left(1+3\phi \right).$$

The advantage of this model is its simplicity. The knowledge of the particle’s thermal conductivity is not a requirement, unlike the Maxwell [14], Hamilton and Crosser [15], and Jeffery [16] models. Although, this advantage comes with a drawback. As the particle’s thermal conductivity approaches the base fluid’s thermal conductivity, the accuracy of the Timofeeva et al. [17] model is expected to decrease. Typically, the particle’s thermal conductivity is significantly greater than that of the base fluid, so in most cases, this would not be an issue.

When testing existing models against Fe_3_O_4_–water nanofluid experimental data, Sundar et al. [18] found that they were not capable of accurately predicting their thermal conductivity measurements. Because of this, Sundar et al. [18] sought to develop a new correlation to accurately predict the thermal conductivity datasets they collected. The Timofeeva et al. [17] model was chosen as a starting point due to its simplicity. From there, the coefficients were modified to support their observations. The data used was within a temperature range of 20–60 °C and a volume fraction range of 0–2%, so the model is only valid within those ranges. The Sundar et al. [18] model is given as:

(8) $$k_{nf}=k_{bf}{\left(1+10.5\phi \right)}^{0.1051}.$$

The advantages and disadvantages of this model are similar to the model of Timofeeva et al. [17] since it follows the same structure.

**Figure 1:**
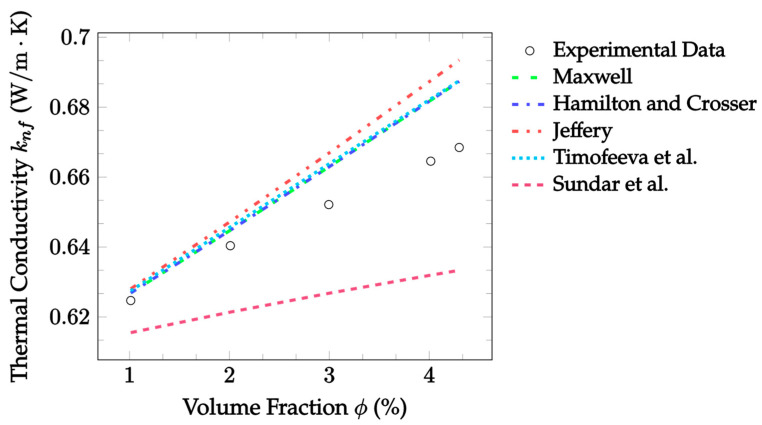
EMT model predictions compared with experimental data for spherical Al_2_O_3_–water nanofluid at 300 K with volume fractions between 1.0–4.3%: Experimental Data [12], Maxwell [14], Hamilton and Crosser [15], Jeffery [16], Timofeeva et al. [17], Sundar et al. [18].

Figure 1 shows EMT model predictions compared to experimental data for Al_2_O_3_–water nanofluid at room temperature as a function of the volume fraction. Due to the particles examined here being spherical, the Maxwell and Hamilton and Crosser models gave identical results. With that in mind, the Maxwell [14] and Hamilton and Crosser [15] models performed best with an MAE of 1.626%, followed closely by the Timofeeva et al. [17] model with an MAE of 1.719% for the given experimental data. In this case, all models except for the Sundar et al. [18] model predicted the thermal conductivity well at low volume fractions but tended to overestimate the enhancement at higher volume fractions. In contrast, Sundar et al. [18] underestimated the thermal conductivity at all temperatures, and additionally underestimated the rate at which the thermal conductivity would change with respect to a changing volume fraction. However, as the model was created exclusively using Fe_3_O_4_ data, it is reasonable to expect the model would not predict the thermal conductivity as accurately for other particle types.

**Figure 2:**
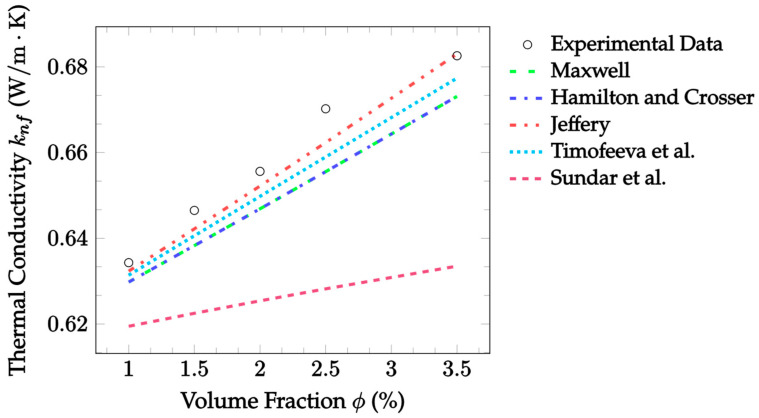
EMT model predictions compared with experimental data for spherical CuO–water nanofluid at 300 K with volume fractions between 1.0–3.5%: Experimental Data [59], Maxwell [14], Hamilton and Crosser [15], Jeffery [16], Timofeeva et al. [17], Sundar et al. [18].

Figure 2 also demonstrates the accuracy of EMT model predictions against the experimental data, now for CuO–water nanofluid. This data was gathered at a room temperature of 300 K, but volume fractions ranged from 1.0–3.5%. In this case, Jeffery [16] best predicted the thermal conductivity, with an MAE of 0.547%, followed by Timofeeva et al. [17] with an MAE of 0.941% for the given experimental data. In this case, all models examined underestimated the thermal conductivity at most volume fractions, with only the Jeffery [16] model reaching the experimental thermal conductivity at a volume fraction of 3.5%. Again, the Sundar et al. [18] model underestimated more severely at all temperatures, but the data examined here was again for a different particle material than that which the model was based on.

Table 1 shows the mean absolute error for each model against several experimental datasets examined. The experimental data came from five different sources and used various particle materials at different volume fractions, and with different base fluids. All data were from nanofluids which used spherical particles, except for one which used CNTs. No individual model consistently outperformed the rest by a significant margin. The Maxwell [14] model had the smallest MAE for two datasets, Hamilton and Crosser [15] for three, Jeffery [16] for six, and Sundar et al. [18] for one. This demonstrates that the Jeffery [16] model consistently had the lowest MAE, but for many cases, the difference in errors was less than 0.5–1%. The models consistently became less accurate as the volume fraction increased. Additionally, the models were generally consistent with each other in regard to which datasets were predicted well and which had substantially higher errors. This was expected, as Hamilton and Crosser [15], Timofeeva et al. [17], and Jeffery [16] all based their models on the work of Maxwell [14]. Meanwhile, Sundar et al. [18] based their model on the work of Timofeeva et al. [17] but used a different particle material that was not seen in the examined independent data. Most datasets used specified spherical particles or did not specify a shape factor (in which case n=3 was assumed, as it was the most common). However, some data for cylindrical carbon nanotubes suspended in water were used. The incorporation of a shape factor in the Hamilton and Crosser [15] model did improve their model compared to the others. Furthermore, the high thermal conductivity of the carbon nanotubes compared to the metal oxides considered when these models were developed caused high error in all models.

### 2.2. Brownian Models

In Effective Medium Theory, nanoparticles are assumed to be stationary within their suspensions. However, it has been shown that particularly at the nanoscale, Brownian motion may have an impact on the bulk thermal conductivity of the nanofluid [41,63,64]. Specifically, it was proposed that collisions between nanoparticles and base fluid molecules, thermal diffusion in nanoparticles within their suspensions, and nanoconvection are important mechanisms when considering the random motion of nanofluid particles [41,64]. It is noted that some findings report the opposite, where Brownian motion has a minor or even negligible effect on the thermal conductivity [33]. Thus, there is still a debate on whether it is useful to consider Brownian motion as a heat transfer mechanism in nanofluids. Even still, models have been developed to predict the nanofluid thermal conductivity with Brownian motion as the primary method of heat transfer. Typically, models that consider Brownian motion have an advantage over EMT models. In comparison, they consider many more effects on the thermal conductivity. In theory, this would make them more accurate at a higher frequency. However, depending on the model, some simplicity can be sacrificed as a result.

Corcione [19] gathered various sets of experimental data from existing literature and performed regression analysis to arrive at a model for the nanofluid thermal conductivity. The data used had property values over a wide range, including particle materials of TiO_2_, CuO, Cu, and Al_2_O_3_, as well as particle diameters from 10 nm to 150 nm, volume fractions between 0.2% and 9%, and temperatures between 294 K and 324 K. Data for nanofluids with both water and ethylene glycol as base fluids were included. Equation (9) gives the Corcione [19] model:

(9) knf=(1+4.4Re0.4Pr0.66(TTfr)10(kpkbf)0.03ϕ0.66)kbf,

where $T_{fr}$ is the freezing temperature of the base fluid in K. The Prandtl number Pr, Reynolds number Re, and Brownian velocity $u_{B}$ are defined by Equations (10)–(12), respectively:

(10) $$\mathrm{Pr}=\frac{c_{bf}\mu_{bf}}{k_{bf}},$$

(11) Re=ρbfuBdpμbf,

(12) $$u_{B}=\frac{2k_{b}T}{\pi \mu_{bf}d_{p}^{2}},$$

where $c_{bf}$ is the specific heat capacity of the base fluid and $k_{b}$ is the Boltzmann constant. It is seen that Brownian motion is considered through the Reynolds number in this case. Unlike the previously discussed EMT models, the temperature, nanoparticle size, and the density, viscosity, and specific heat of the base fluid are all considered by Corcione [19]. This model also has a broader validity range, making it better for engineering applications. While the properties considered are more involved, they have been extensively researched and are typically not difficult to determine. Unlike the Hamilton and Crosser [15] model, however, particle shape is not accounted for.

Chon et al. [20] also formulated a model with Brownian motion in mind. Al_2_O_3_–water nanofluids were produced with equivalent particle diameters of 11 nm, 47 nm, and 150 nm, and with volume fractions of 1% and 4%. Thermal conductivity measurements across a temperature range of 21 to 71 °C were taken and used in combination with the Buckingham π Theorem to establish a new model, shown in Equation (13):

(13) knf=(1+64.7ϕ0.746(dbfdp)0.369(kpkbf)0.746Pr0.9955Re1.2321)kbf,

where Pr is defined as shown in Equation (10), and Re is defined as:

(14) Re=ρbfkbT3πμbf2lbf.

Once again, Brownian motion is considered through the Reynolds number, which is the highest order factor within the model. The Chon et al. [20] model differs from the Corcione [19] model in that it has a much narrower validity range. This could be advantageous in cases where Al_2_O_3_–water nanofluid is used, as the empirical constants would be tuned for that case. It is also a disadvantage since it will likely be less accurate for nanofluids with other particle and base fluid materials. Temperature is also not taken into consideration as a direct parameter, unlike the model by Corcione [19].

Continuing the work done by Maxwell [14], a new model was developed by Koo and Kleinstreuer [21]. It was theorized that the thermal conductivity improvement due to Brownian motion was additive to the thermal conductivity of an equivalent, stationary nanofluid. That is, $k_{stationary}+k_{Brownian}=k_{nf}$, where $k_{stationary}$ is the thermal conductivity predicted by the Maxwell [14] model in Equation (2). An equation for the Brownian thermal conductivity term was derived by considering the particle interactions between two nanoparticles. In addition to the Corcione [19] and Chon et al. [20] models, Koo and Kleinstreuer [21] also considered the effect of nanoparticle movement on the base fluid particles. It was believed that the nanoparticles would carry some amount of surrounding base fluid with them, thus acting as an agent for micromixing to take place. This phenomenon would also work to enhance the thermal conductivity of the nanofluid. With the additional use of the existing experimental data, the resulting equation is the Koo and Kleinstreuer [21] model, given in Equation (15):

(15) $$k_{nf}=\left(\frac{k_{p}+2k_{bf}-2\phi \left(k_{bf}-k_{p}\right)}{k_{p}+2k_{bf}+\phi \left(k_{bf}-k_{p}\right)}+5\times 10^{4}\beta \phi \rho_{bf}c_{bf}\sqrt{\frac{k_{b}T}{\rho_{p}d_{p}}}f\left(T,\phi \right)\right)k_{bf},$$

where β is an empirical correlation factor, and f(T,ϕ) accounts for the temperature dependence of the thermal conductivity. β was assumed to be solely a function of the volume fraction and nanoparticle and is defined in four separate cases in the temperature range of 300 K ≤T≤ 325 K. First, $\beta =0.0011{\left(100\phi \right)}^{-0.7272}$ for CuO nanoparticles and ϕ>1% [21]. However, if ϕ<1%, $\beta =0.0137{\left(100\phi \right)}^{-0.8229}\;$[21]. If the nanoparticle material is Al_2_O_3_ and ϕ>1%, then $\beta =0.0017{\left(100\phi \right)}^{-0.0841}\;$[21]. Furthermore, if the nanoparticle material is either Au-citrate or Ag-citrate and ϕ<1%, then $\beta =0.0137{\left(100\phi \right)}^{-0.8229}\;$[21].

The function f(T,ϕ) was also found using existing data, and is given in Equation (16) with a validity range of 1% ≤ ϕ≤ 4% and 300 K≤T≤325 K:

(16) f(T,ϕ)=(−6.04ϕ+0.4705)T+(1722.3ϕ−134.63).

The main advantage of using the Koo and Kleinstreuer [21] model is that it considers Brownian motion more directly than the previously outlined models. It also not only considers the interactions between nanoparticles, but those between nanoparticles and base fluid molecules. There is an increase in complexity, however. The empirical correlation β has various definitions based on the particle material and concentration used. This may be inconvenient for application, especially when the nanofluid considered does not align with any of the various definitions.

In the pursuit of determining a model that best fits their data, Vajjha and Das [22] expanded upon the Koo and Kleinstreuer [21] model. Experiments were conducted on Al_2_O_3_, CuO, and ZnO–EG–water nanofluids at temperatures ranging from 298 K to 363 K, with particle diameters of 29 nm, 53 nm, and 77 nm used. It was observed that the Koo and Kleinstreuer [21] model had the best agreement with the given experimental data; however, the range of validity was too narrow. Thus, using the 133 thermal conductivity datapoints obtained, new correlations for f(T,ϕ), shown in Equation (17), and β were derived:

(17) $$f\left(T,\phi \right)=\left(0.028217\phi +0.003917\right)\left(\frac{T}{T_{0}}\right)-\left(0.030669\phi +0.00391123\right),$$

where the reference temperature $T_{0}=273.15\;K$. Vajjha and Das [22] defined β in three cases within a temperature range of 298 K≤T≤363 K. Given a nanoparticle material of CuO and 1%≤ϕ≤6%, $\beta =9.881{\left(100\phi \right)}^{-0.9446}\;$[22]. In the case of ZnO or Al_2_O_3_ nanoparticles in concentrations of 1%≤ϕ≤7% or 1%≤ϕ≤10%, respectively, then $\beta =8.4407{\left(100\phi \right)}^{-1.07304}$ [22].

Given that the model is heavily based on the work of Koo and Kleinstreuer [21], it has similar advantages and disadvantages. However, it has an additional advantage over the work of Koo and Kleinstreuer [21]. Specifically, it has a wider validity range, which makes it applicable to more nanofluids.

Furthermore, Patel et al. [23] experimented with Al_2_O_3_, CuO, Cu, and Al nanofluids using water, ethylene glycol, and transformer oil as base fluids. The parameters varied included the volume fraction, particle diameter, and temperature. Non-linear regression analysis was performed on the obtained data, and the resulting Patel et al. [23] model is given in Equation (18):

(18) $$k_{nf}=\left(1+0.135{\left(\frac{k_{p}}{k_{bf}}\right)}^{0.273}\phi^{0.467}{\left(\frac{T}{20}\right)}^{0.547}{\left(\frac{100}{d_{p}}\right)}^{0.234}\right)k_{bf},$$

where T is expressed in °C, and $d_{p}$ is expressed in nm. The claimed range of validity of this model is for spherical nanoparticles 10 to 150 nm in diameter with thermal conductivities between 20 and 400 W/m·K, base fluid thermal conductivities between 0.1 and 0.7 W/m·K, volume fractions from 0.1% to 3%, and temperatures from 20 °C to 50 °C. It was proposed that Brownian motion was accounted for through the particles’ diameters. This was concluded from their discovery that a temperature increase provided greater enhancement in nanofluids with smaller particle diameters. Specifically, the Brownian velocity of the 150 nm particles was found to be two orders of magnitude lower than that of the 11 nm particles [23]. Therefore, when increasing the temperature and consequently decreasing the base fluid’s viscosity, the increase in Brownian velocity would be more pronounced in the smaller particles [23]. In contrast to the other Brownian models, Patel et al. [23] were able to obtain a much higher validity range overall. Its complexity is also significantly reduced compared to the Koo and Kleinstreuer [21] and Vajjha and Das [22] models. It is, however, purely an empirical correlation. It does not directly consider Brownian motion through a heat transfer model.

Finally, Kumar et al. [24] derived an equation for thermal conductivity by considering the Brownian motion of the nanoparticles directly. Similar to what Patel et al. [23] observed, Kumar et al. [24] believed the strong temperature dependence of the thermal conductivity is due to the increase in the Brownian velocity of the nanoparticles. Therefore, Brownian motion was accounted for by applying a dynamic model to a separate static model. The dynamic model is expressed as $k_{p}=C_{u}u_{B}$, where $C_{u}$ is a constant. Thus, the Kumar et al. [24] model is shown below:

(19) $$k_{nf}=\left(1+C_{u}u_{B}\frac{\phi r_{bf}}{k_{bf}\left(1-\phi \right)r_{p}}\right)k_{bf},$$

where $u_{B}$ is defined as shown in Equation (12). In this paper, it is assumed that $C_{u}=2.95$ since Kumar et al. [24] found that a value between 2.9 and 3 worked for some datasets, and no explicit value was given. The Kumar et al. [24] model is the simplest of the Brownian models discussed [19,20,21,22,23], which is its primary advantage. In addition, like the Chon et al. [20] model, it does consider the size of the base fluid’s molecules. In some cases, this may be a difficult parameter to find. However, for common base fluids, this value is typically known. The main disadvantage with this model is that it does not directly consider the particles’ thermal conductivity, like the Timofeeva et al. [17] and Sundar et al. [18] EMT models. The constant $C_{u}$ is also not known for many nanofluids, which is another disadvantage.

**Figure 3:**
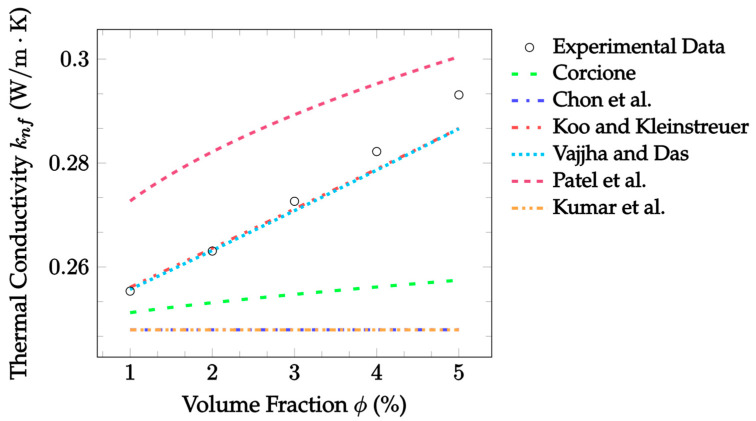
Brownian model predictions compared with experimental data for Al_2_O_3_–EG nanofluid at 300 K, with a particle diameter of 24.4 nm and volume fractions from 1% to 5%: Experimental Data [12], Corcione [19], Chon et al. [20], Koo and Kleinstreuer [21], Vajjha and Das [22], Patel et al. [23], Kumar et al. [24].

In Figure 3, the accuracy of the Brownian models when measured against experimental data for Al_2_O_3_–EG nanofluid, is visualized. It can be seen that the Koo and Kleinstreuer [21] and Vajjha and Das [22] models performed the best, with mean absolute errors of 0.884% and 0.867%, respectively. The Patel et al. [23] model slightly overestimated the thermal conductivity, and thus resulted in a higher mean absolute error of 5.456%. The remaining three models all underestimated thermal conductivity improvements with respect to the volume fraction, especially Chon et al. [20] and Kumar et al. [24], which predicted a constant thermal conductivity.

**Figure 4:**
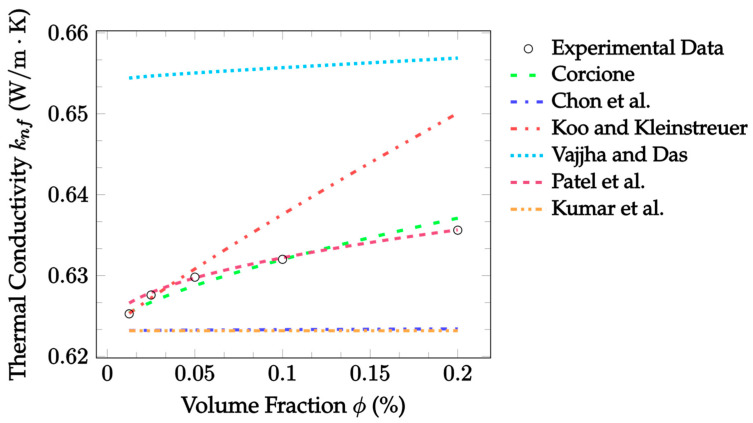
Brownian model predictions and experimental findings for ZrO_2_–water nanofluid at 35 °C, with a particle diameter of 30 nm and volume fractions from 0.0125% to 0.2%: Experimental Data [65], Corcione [19], Chon et al. [20], Koo and Kleinstreuer [21], Vajjha and Das [22], Patel et al. [23], Kumar et al. [24].

Figure 4 shows Brownian model predictions for ZrO_2_–water nanofluid, along with experimental data gathered by Çolak [65] for significantly low volume fractions. The Patel et al. [23] model has exceptional results in this case. It gave a near exact prediction of the non-linear improvement in thermal conductivity, except for a slight overestimate at ϕ = 0.0125%. The mean absolute error was 0.0624%, which is relatively nice. The Corcione [19] model was also in good agreement with this dataset, having a mean absolute error of 0.114%. Regarding the Koo and Kleinstreuer [21] and Vajjha and Das [22] models, β was undefined for ZrO_2_ nanoparticles. So, it was assumed β as defined for CuO particles could be applied, since their thermal conductivity values were approximately the same. As expected, these models did not perform as well as in the previous case, and thus, it is advised to only apply the model when the proper particle materials are in use. Lastly, the Chon et al. [20] and Kumar et al. [24] models predicted a constant thermal conductivity with respect to the volume fraction for the second time. So, the models’ accuracy for given experimental data as a function of temperature should be determined.

**Figure 5:**
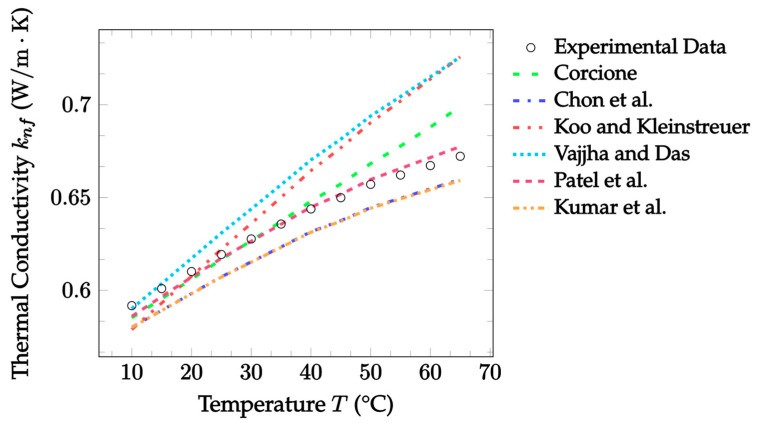
Brownian models as a function of temperature compared with experimental data gathered for ZrO_2_–water with 30 nm particle diameter, volume fraction of 0.2%, and temperatures ranging from 10 °C to 65 °C: Experimental Data [65], Corcione [19], Chon et al. [20], Koo and Kleinstreuer [21], Vajjha and Das [22], Patel et al. [23], Kumar et al. [24].

In Figure 5, thermal conductivity is shown as a function of temperature for the Brownian models and more ZrO_2_–water data [65]. Once again, the Patel et al. [23] model performs the best in this case, with a mean absolute error of 0.461%. The Koo and Kleinstreuer [21] and Vajjha and Das [22] models are able to accurately predict thermal conductivity at low temperatures, but less so at higher temperatures which are outside their validity ranges. Again, this was expected and reasonable. Lastly, the Chon et al. [20] and Kumar et al. [24] models both successfully predict an increase in thermal conductivity with an increase in temperature. Thus, these models are best suited for datasets where temperature is the independent variable.

**Figure 6:**
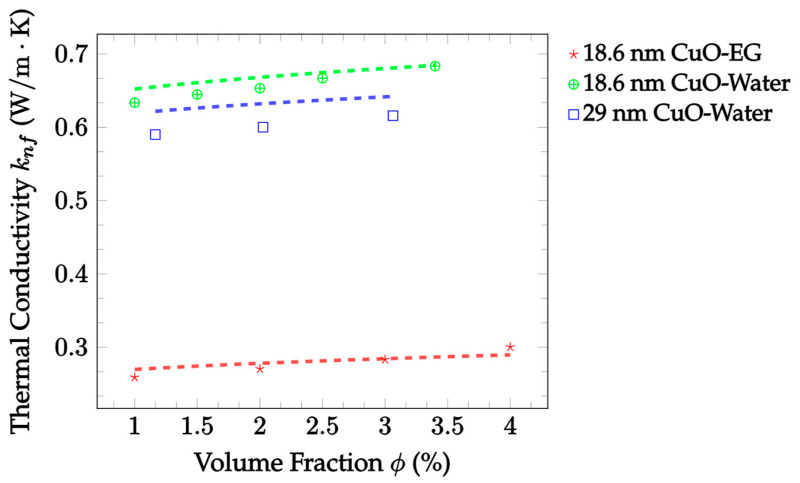
Patel et al. [23] model compared with experimental data for nanofluids with higher particle concentrations: 18.6 nm CuO–EG [12], 18.6 nm CuO-Water [12], 29 nm CuO–water [66].

Currently, the Patel et al. [23] model has consistently predicted experimental findings with a high level of accuracy. Therefore, more predictions compared with experimental data are shown in Figure 6 to further illustrate this trend. The data have a fixed particle material of CuO to allow for a more accurate investigation of the other parameters. Collectively, the data show that the Patel et al. [23] model is capable throughout and slightly beyond its validity range for the concentration. The same can be said for most of the range of valid base fluids’ thermal conductivities. Of these cases, the model has the highest level of accuracy when predicting the 18.6 nm CuO–water nanofluid, yielding an error of 1.804%. This is then followed by slightly higher errors of 2.719% and 4.997% for the CuO–EG and 29 nm CuO–water nanofluids, respectively. In addition, it is noted that the model had somewhat lower accuracy for nanofluids with larger nanoparticles. This set of data is from a different source, however, so more data are needed to confirm if this is true in general.

In Table 2 below, the mean absolute error for each model in each case is shown. Introduced datasets were found from three separate sources, all with varying particle diameters, particle materials, base fluids, volume fractions, and temperatures. The Patel et al. [23] model performed the best for six of the eleven studied, the highest of any of the Brownian models. It also had the smallest range of error at 5.394%, making it the most precise as well as the most accurate. The next best model was found to be the Corcione [19] model, performing the best for three of the eleven sets. In general, all models performed well at sufficiently low volume fractions. The one exception is the Vajjha and Das [22] model in the 65 °C ZrO_2_–water dataset, but this only shows that the model becomes less accurate when approaching temperatures of 65 °C or higher. It still has a good performance when the temperature is within its validity range. Despite the prediction of a constant thermal conductivity with a changing volume fraction by the Chon et al. [20] and Kumar et al. [24] models, their accuracy is on par with the remaining models investigated. It was also observed that the Chon et al. [20] model performed best with Al_2_O_3_–water data, which was predicted upon its introduction.

### 2.3. Nanolayer Models

The next mode of thermal conductivity enhancement considered by the existing literature has to do with the presence of the nanolayer. The nanolayer is an interfacial layer that exists between the solid nanoparticle and the surrounding base fluid [42]. Yu et al. [67,68] found experimentally that it has a highly ordered molecular structure. So, it would have thermal properties similar to those found in crystalline solids, including a higher thermal conductivity. Currently it is known that the nanolayer’s thickness is on the order of nanometers [69]. Beyond this, not much more is known about its characteristics. Thus, various assumptions are typically made when applying nanolayer models. Leong et al. [25] showed that the thermal conductivity of the nanolayer could be estimated to be two to three times that of the base fluid’s thermal conductivity through an analysis of experimental data. Therefore, in this paper, it was assumed for all nanolayer calculations that $k_{nl}=2k_{bf}$, and that the nanolayer’s thickness $\delta_{nl}=1$ nm.

Leong et al. [25] developed a model for the nanofluid thermal conductivity considering the effects of the interfacial layer. It was derived from the general, cylindrical two-dimensional heat conduction equation, assuming spherical non-interacting particles and a steady-state heat transfer. The nanofluid was broken down into three fundamental parts, namely, the nanoparticle, nanolayer, and surrounding base fluid. Equal temperature and heat conduction boundary conditions were specified at both sides of the interface. Once again, the model was compared to experimental data, and it was found that $\delta_{nl}=1$ nm and $k_{nl}=2k_{bf}$ were acceptable values. The Leong et al. [25] model is shown below:

(20) $$k_{nf}=\frac{\left(k_{p}-k_{nl}\right)\phi k_{nl}\left[2{\left(1+\frac{\delta_{nl}}{2r_{p}}\right)}^{3}-{\left(1+\frac{\delta_{nl}}{r_{p}}\right)}^{3}+1\right]+\left(k_{p}+2k_{nl}\right){\left(1+\frac{\delta_{nl}}{2r_{p}}\right)}^{3}\left[\phi {\left(1+\frac{\delta_{nl}}{2r_{p}}\right)}^{3}\left(k_{nl}-k_{bf}\right)+k_{bf}\right]}{{\left(1+\frac{\delta_{nl}}{2r_{p}}\right)}^{3}\left(k_{p}+2k_{nl}\right)-\left(k_{p}-k_{nl}\right)\phi \left[{\left(1+\frac{\delta_{nl}}{2r_{p}}\right)}^{3}+{\left(1+\frac{\delta_{nl}}{r_{p}}\right)}^{3}-1\right]},\;$$

where $k_{nl}$ is the thermal conductivity of the nanolayer, $\delta_{nl}$ is the nanolayer’s thickness, and $r_{p}$ is the nanoparticle radius. The Leong et al. [25] model does not use empirical constants, so there are no validity range issues. This gives it an advantage over all the Brownian models discussed. It utilizes the same assumptions as Maxwell [14], assuming spherical, non-interacting particles. However, it additionally considers the particle size, giving it an advantage over all the EMT models discussed. Nonetheless, the model is significantly more complex than the EMT or Brownian models analyzed. This is due to the use of the nanolayer properties, which are not well known in the literature. This would make it more difficult to apply in many scenarios.

Xie et al. [26] also derived a nanofluid thermal conductivity model based on the nanolayer. It was presumed that its effects are significant at the nanoscale since the specific surface area of nanoparticles are approximately one thousand times greater than that of micro-scale particles. It was also believed that the nanolayer’s conductivity is a function of the particle and base fluid’s conductivities, as well as the nanolayer’s size relative to the particle. The model was derived using Fourier’s law of heat conduction, assuming that the nanolayer’s thermal conductivity varied linearly with the nanolayer’s thickness and steady-state heat transfer. The resulting model was simplified further by performing an order of magnitude analysis and considering sufficiently low volume fractions. At last, the Xie et al. [26] model is presented in Equation (21):

(21) $$k_{nf}=k_{bf}\left(3\Theta \phi +\frac{3\Theta^{2}\phi^{2}}{1-\Theta \phi }+1\right),$$

where Θ is defined in Equation (22):

(22) $$\Theta =\frac{\left(\frac{k_{nl}-k_{bf}}{k_{nl}+2k_{bf}}\right)\left[{\left(1+\frac{\delta_{nl}}{r_{p}}\right)}^{3}-\left[\frac{\left(k_{p}-k_{nl}\right)\left(k_{bf}+2k_{nl}\right)}{\left(k_{p}+2k_{nl}\right)\left(k_{bf}-k_{nl}\right)}\right]\right]}{{\left(1+\frac{\delta_{nl}}{r_{p}}\right)}^{3}+2\left[\left(\frac{k_{nl}-k_{bf}}{k_{nl}+2k_{bf}}\right)\left(\frac{k_{p}-k_{nl}}{k_{p}+2k_{nl}}\right)\right]}.$$

Like the work of Leong et al. [25], Xie et al. [26] did not use empirical analysis to find any unknown constants, which is a significant advantage. Although, due to the order of magnitude analysis, the concentration of the nanofluids it can be applied to is arbitrarily limited.

More research was done by Yu and Choi [27], who modified the Maxwell [14] model to incorporate the enhancement effects of the nanolayer. A similar approach used by Leong et al. [25] was taken, where the effects of the nanoparticles and the nanolayer were combined into an equivalent particle. This resulting particle has an increased diameter and volume concentration in the fluid. Here, the issue arises where the collisions or near-collisions of the smaller, actual particles result in the equivalent particles partially ”phasing” through each other. Hence, it was necessary to assume that the particle concentration is low enough such that this scenario does not occur. It was also assumed that the particles were spherical and of uniform size. Substituting the properties of the equivalent particle into the Maxwell [14] model yielded the following equation:

(23) $$k_{nf}=\left(\frac{k_{pe}+2k_{bf}+2\left(k_{pe}-k_{bf}\right){\left(\beta_{nl}\right)}^{3}\phi }{k_{pe}+2k_{bf}-\left(k_{pe}-k_{bf}\right){\left(\beta_{nl}\right)}^{3}\phi }\right)k_{bf},$$

where $k_{pe}$ and $\beta_{nl}$, defined in Equations (24) and (25), are the thermal conductivity of the equivalent particle and the factor that converts particle concentration to the equivalent particle concentration, respectively. Consider that $\gamma =k_{nl}/k_{p}$ in Equation (24) is merely the ratio of the nanolayer’s thermal conductivity to that of the particle.

(24) $$k_{pe}=\left(\frac{\left[2\left(1-\gamma \right)+{\left(\beta_{nl}\right)}^{3}\left(1+2\gamma \right)\right]\gamma }{-\left(1-\gamma \right)+{\left(\beta_{nl}\right)}^{3}\left(1+2\gamma \right)}\right)k_{p},$$

(25) $$\beta_{nl}=1+\frac{\delta_{nl}}{r_{p}}.$$

Since the Yu and Choi [27] model is similar to the Leong et al. [25] and Xie et al. [26] models, it has almost similar advantages and disadvantages.

Finally, an early model developed by Tinga et al. [28] to predict the complex dielectric constant of a multiphase mixture was adapted to predict the thermal conductivity of nanofluids, just as the Maxwell [14] model was. Originally, the model was inspired by a mixture of air, water, and cellulose, analogous to the base fluid, nanolayer, and nanoparticles in a nanofluid, respectively. A reduced version of the general model for spherical inclusions, or nanoparticles, was used. The resulting model for the nanofluid thermal conductivity is given below:

(26) $$k_{nf}=\left(1+\frac{3\phi \left[\left(\beta_{nl}{}^{3}-1\right)\left(2k_{nl}+k_{p}\right)\left(k_{nl}-k_{bf}\right)-\left(k_{nl}-k_{p}\right)\left(2k_{nl}+k_{bf}\right)\right]}{\left(2k_{bf}+k_{nl}\right)\left(2k_{nl}+k_{p}\right)-\left(\frac{2}{\beta_{nl}{}^{3}-1}\right)\left(k_{nl}-k_{bf}\right)\left(k_{nl}-k_{p}\right)-3\phi k_{nl}\left(k_{p}-k_{bf}\right)}\right)k_{bf}.$$

**Figure 7:**
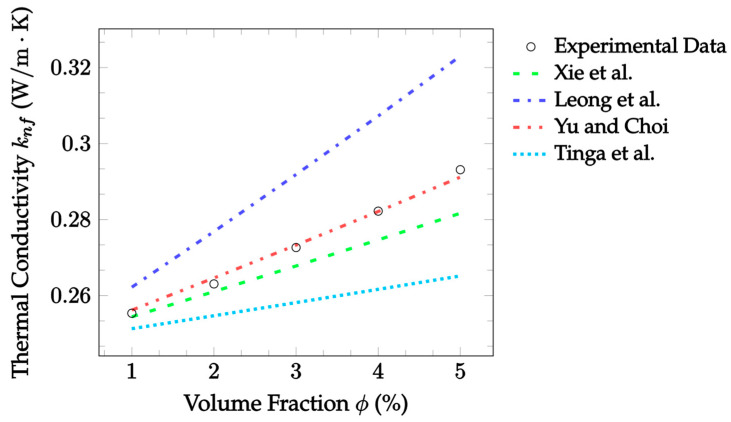
Nanolayer nanofluid thermal conductivity predictions compared with data for Al_2_O_3_–EG nanofluid at 27 °C and with a particle diameter of 24.4 nm: Experimental Data [12], Xie et al. [26], Leong et al. [25], Yu and Choi [27], Tinga et al. [28].

In Figure 7, the theoretical predictions of the nanolayer models are compared against experimental data for Al_2_O_3_–EG nanofluid. In this case, the Yu and Choi [27] model is in excellent agreement with observations. It was able to correctly predict the rate of enhancement along with the initial value, thus resulting in a low MAE of 0.369%. The Xie et al. [26] model also did well in predicting this set of data. It only slightly underestimated the thermal conductivity overall, and hence, a moderately higher error of 1.900% compared with the Yu and Choi [27] model was found. The remaining Leong et al. [25] and Tinga et al. [28] models tended to overestimate and underestimate the thermal conductivity, respectively. Even still, they gave relatively low errors of 6.808% and 5.382%, meaning, overall, that the nanolayer models were successfully able to predict this dataset.

The level of accuracy of the nanolayer models when compared against experimental observations is shown in Table 3 below. Each model was applied to datasets of differing temperature, particle material and size, and base fluid. In general, the Leong et al. [25], Yu and Choi [27], and Tinga et al. [28] models performed similarly, each giving the best predictions for three of the ten datasets examined. However, it was noted that the observations by Mintsa et al. [66] were not accurately predicted by any of the models except for the Tinga et al. [28] model. As a result, this model was the most precise of the ones analyzed, with an error range of 4.714%. Lastly, while the Xie et al. [26] model only performed best for one set of data, it still easily competed with the rest of the nanolayer models.

### 2.4. Aggregation Models

Every model discussed so far has in some form assumed the nanoparticles to be well-dispersed and non-interacting within their suspensions. However, it has been found experimentally with distinct methodologies that this is not the case [71]. In general, aggregation occurs due to collisions brought about by inevitable particle transportation. A fraction of these collisions can then be totally inelastic due to existing inter-particle forces [43]. There is a disagreement among researchers on whether nanoparticle aggregation improves or worsens the heat transferring abilities of nanofluids. For example, it is reported that aggregation could improve thermal conductivity by creating low resistance paths along the clusters [44], and it is also predicted that the reduction in Brownian motion would lower thermal conductivity [30]. This disagreement is evident in some of the model predictions. A typical approach for aggregate models is to describe clusters in terms of fractal geometry, shown to be viable by Meakin [72]. So, when necessary, this paper assumes the fractal dimension $d_{f}=1.8$ per the findings of Wang et al. [32]. In addition, nanolayer parameters are also used in some models, so the same assumptions used for calculations in Section 2.3 were applied. This brings up the same concern as with the nanolayer models. The use of nanolayer and aggregate characteristics whose values are not well known means many assumptions are required. In addition, the large number of parameters required to fully describe a single nanofluid here are difficult to find in the existing literature. Consequentially, there are only a few datasets that were found to be applicable to these models.

Prasher et al. [29] formulated a model for the nanofluid thermal conductivity considering the Brownian motion and aggregation of the nanoparticles. It was thought that these two modes of thermal conductivity enhancement are inseparable due to the close relationship between the two. The aggregation process was modeled as time dependent. The suspension was defined as totally dispersed initially, before a single large cluster is formed after an infinite length of time. This aggregation model was then merged with the Maxwell [14] model in Equation (2). The particle volume fraction and thermal conductivity were both replaced with the aggregation volume fraction and thermal conductivity, respectively. In addition, it was assumed that the nanoparticles were spherical and uniform in size. The model is given in Equation (27) below:

(27) $$k_{nf}=\left[\frac{k_{a}+2k_{bf}+2\phi_{a}\left(k_{a}-k_{bf}\right)}{k_{a}+2k_{bf}-\phi_{a}\left(k_{a}-k_{bf}\right)}\right]k_{bf},\;$$

where $k_{a}$ is the thermal conductivity of the aggregates, expressed in Equation (28), and $\phi_{a}$ is the volume fraction of the aggregates, expressed in Equation (29):

(28) $$k_{a}={\left(\frac{r_{a}}{r_{p}}\right)}^{d_{f}-3}\left(k_{p}-k_{bf}\right)+k_{bf},$$

(29) $$\phi_{a}=\frac{\phi }{{\left(r_{a}/r_{p}\right)}^{d_{f}-3}}.$$

In both expressions, $r_{a}$ is the radius of gyration of the aggregates (radius of the smallest disk encompassing an entire cluster), and $d_{f}$ is the fractal dimension. This model predicts that if the aggregates remain well-dispersed, an increase in aggregation could enhance the nanofluid thermal conductivity. The primary advantage of this model is that it considers the EMT, Brownian motion, and aggregation enhancement mechanisms simultaneously. However, it also requires the knowledge of the fractal dimension of the aggregates. Knowledge of this parameter is greatly limited in literature, and thus, the model cannot be applied without significant assumptions.

Xuan et al. [30] also devised a model, again considering both Brownian motion and aggregation of the nanoparticles. In this case, aggregation was modeled using a simulation. However, unlike Prasher et al. [29], the maximum cluster size was artificially limited. From here, a methodology similar to that used by Koo and Kleinstreuer [21] was applied. Xuan et al. [30] believed that the enhancement due to Brownian motion and aggregation was additive to the enhancement predicted by EMT. Here, the thermal conductivity of the stationary fluid was also predicted using the Maxwell [14] model in Equation (2). The proposed model is given as:

(30) $$k_{nf}=\left(\frac{k_{p}+2k_{bf}+2\phi \left(k_{p}-k_{bf}\right)}{k_{p}+2k_{bf}-\phi \left(k_{p}-k_{bf}\right)}+\frac{\rho_{p}\phi c_{p}}{2k_{bf}}\sqrt{\frac{k_{B}T}{3\pi r_{a}\mu_{bf}}}\right)k_{bf},$$

where $c_{p}$ is the specific heat of the nanoparticle in J/kg·K, and T is measured in K. This model predicts that as the size of the aggregates increases, the thermal conductivity enhancement decreases. This is the only aggregation model that does not require the knowledge of complicated parameters. Therefore, in situations where aggregation may be a primary enhancement mechanism, the Xuan et al. [30] model may be the best approach. The most difficult value to measure in this model is the radius of the aggregates, but this is something that has been found from experimentation [73,74,75,76]. In addition, Xuan et al. [30] account for the EMT, Brownian motion, and aggregation enhancement mechanisms. However, a different approach is taken than that used by Prasher et al. [29].

Another model was proposed by Feng et al. [31] with aggregation and the nanolayer as the main mechanisms of thermal conductivity enhancement. The work done by Yu and Choi [27] was expanded upon by incorporating the aggregation of nanoparticles, since the original model only applied to well-dispersed suspensions. This was completed by using a weighted sum of the particle and aggregate conductivities with respect to their volume fractions. Aggregation was modeled by considering a portion of a two-dimensional lattice of touching, spherical nanoparticles. Then, the total thermal resistance of the cluster was found using a one-dimensional thermal network. In combination with Fourier’s law, an expression for the thermal conductivity of the nanofluid could be found. The resulting model by Feng et al. [31] is expressed in Equation (31):

(31) $$k_{nf}=\left(1-\phi_{e}\right)k_{m}+\phi_{e}\left[\left(1-\frac{3}{2}\phi_{e}\right)k_{bf}+\frac{3\phi_{e}k_{bf}}{\alpha }\left(\frac{1}{\alpha }\ln\frac{r_{p}+\delta_{nl}}{\left(r_{p}+\delta_{nl}\right)\left(1-\alpha \right)}-1\right)\right],$$

where $k_{m}$ is the thermal conductivity of the nanofluid calculated by the Yu and Choi [27] model in Equation (23), and $\phi_{e}$ and α are defined in Equations (32) and (33), respectively:

(32) $$\phi_{e}=\phi {\left(\beta_{nl}\right)}^{3},$$

(33) $$\alpha =1-\frac{k_{bf}}{k_{pe}}.$$

Note that $\beta_{nl}$ is defined the same as it was previously in Equation (25). A closer look at Equation (31) reveals that the first term on the right-hand side accounts for the particle conductivity of the non-aggregated nanoparticles, whereas the second term accounts for the aggregates. The Feng et al. [31] model requires the use of nanolayer characteristics, which puts it at a disadvantage compared to the other aggregation models [29,30,32]. However, it does not require the use of the fractal dimension, which is an advantage over the models of Prasher et al. [29] and Wang et al. [32].

At last, Wang et al. [32] produced a model for the nanofluid thermal conductivity with the effects of aggregation considered, based on a modified Maxwell [14] model and the Bruggeman [77] model. Like the model proposed by Yu and Choi [27], this model utilizes fractal geometry to characterize the nanoparticle clusters. It was thought that the distribution of aggregate sizes within the suspension could be expressed using a logarithmic normal distribution function, assuming the geometric mean of the aggregate radius could be replaced with the average nanoparticle radius. It was also assumed the standard deviation of the aggregate size could be taken as a standard value of 1.5. The proposed model by Wang et al. [32] is given in Equation (34):

(34) $$k_{nf}=\left(\frac{1-\phi +3\phi \int_{0}^{\infty }\frac{k_{cl}\left(r_{a}\right)n\left(r_{a}\right)}{k_{cl}\left(r_{a}\right)+2k_{bf}}dr_{a}}{1-\phi +3\phi \int_{0}^{\infty }\frac{k_{bf}n\left(r_{a}\right)}{k_{cl}\left(r_{a}\right)+2k_{bf}}dr_{a}}\right)k_{bf}.$$

The simplified log normal distribution function, as a function of the aggregate radius of gyration, is expressed below:

(35) $$n\left(r_{a}\right)=\frac{1}{r_{a}\sqrt{2\pi }\ln\left(1.5\right)}\exp\left[-{\left(\frac{\ln\left(r_{a}/r_{p}\right)}{\sqrt{2\pi }\ln\left(1.5\right)}\right)}^{2}\right].$$

The expression for $k_{cl}\left(r_{a}\right)$ in Equation (36) was derived from the Bruggeman [77] model, and gives the thermal conductivity of a cluster for a given size:

(36) $$k_{cl}\left(r_{a}\right)=\left(3f\left(r_{a}\right)-1\right)k_{p}+\left[3\left(1-f\left(r_{a}\right)\right)-1\right]k_{bf}+\sqrt{\Delta },$$

where *∆* can be found as:

(37) $$\begin{array}{cc} \Delta ={\left(3f\left(r_{a}\right)-1\right)}^{2}k_{p}^{2} & +{\left[3\left(1-f\left(r_{a}\right)\right)-1\right]}^{2}k_{bf}^{2}\\ & +2\left[2+9f\left(r_{a}\right)\left(1-f\left(r_{a}\right)\right)\right]k_{p}k_{bf}. \end{array}$$

Finally, the function $f\left(r_{a}\right)$ represents the volume fraction of particles in a cluster for a given cluster size, and replaces ϕ in the original Bruggeman [77] model. It is given in Equation (38):

(38) $$f\left(r_{a}\right)={\left(\frac{r_{a}}{r_{p}}\right)}^{d_{f}-3}.$$

Unlike the Prasher et al. [29] and Xuan et al. [30] models, the model by Wang et al. [32] does not consider aggregation as a time-dependent phenomenon. Instead, it assumes there exists a steady distribution of aggregates with varying sizes, which may be an advantage. For instance, a suitably well-dispersed nanofluid may have negligible aggregation over time. In addition, most nanofluids are not totally dispersed. There typically exists some distribution of aggregate sizes within them. The downside comes from the difficulty of describing this distribution and the aggregate shape. There is no way to measure many of the required parameters, and thus, many assumptions are necessary for this model to be applicable.

**Figure 8:**
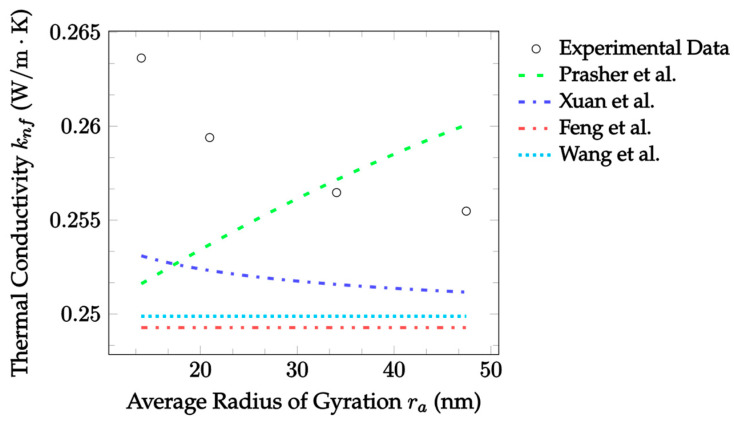
Aggregation model thermal conductivity predictions compared with data for CuO––EG nanofluid: Experimental Data [73], Prasher et al. [29], Xuan et al. [30], Feng et al. [31], Wang et al. [32].

In Figure 8, the theoretical predictions of the aggregation models are compared with experimental data for CuO–EG nanofluid as a function of aggregate size. It was kept at a temperature of 25 °C and had a particle diameter of 10 nm, a volume fraction of 0.18%, and an average aggregate radius of gyration ranging from approximately 13.9 nm to 47.5 nm as time passed for one hour [73]. Firstly, the Prasher et al. [29] model was technically the most accurate, with a mean absolute error of 2.204%. However, it predicts an increase in thermal conductivity with an increase in aggregate size, which does not agree with the experimental results. The Xuan et al. [30] model, while having a slightly lower mean absolute error of 2.578%, correctly predicts a non-linear decrease in thermal conductivity with aggregate size. The Feng et al. [31] and Wang et al. [32] models both predict a constant thermal conductivity with respect to aggregate size, but this is because they both consider aggregation as a process independent of time. It is seen that as the aggregate size increases, which corresponds to time passing in the original experiment, the data approach the values predicted by both models. Thus, these models may be best applied after sufficient time has been provided for the nanoparticles to aggregate.

**Figure 9:**
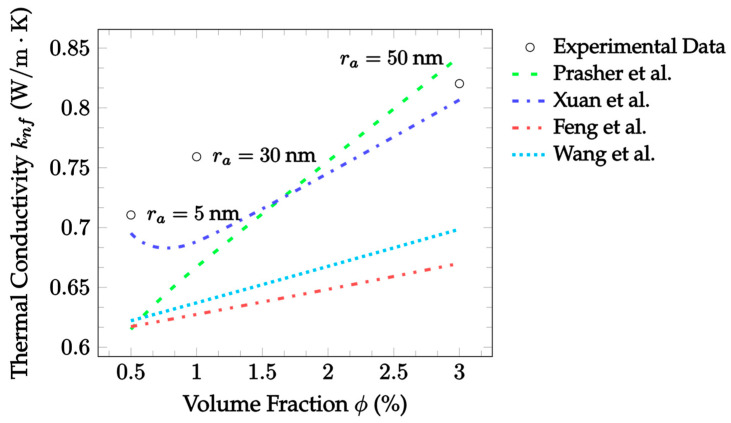
Aggregation model thermal conductivity predictions compared with data for Fe_3_O_4_–water nanofluid: Experimental Data [74], Prasher et al. [29], Xuan et al. [30], Feng et al. [31], Wang et al. [32].

Figure 9 displays the aggregation model predictions compared with more experimental data as a function of the volume fraction. The data were gathered from Fe_3_O_4_–water nanofluid at a temperature of around 25 °C, with a particle diameter of 9.8 nm, volume fraction ranging from 0.5% to 3%, and aggregate radius ranging from 5 nm to approximately 50 nm [74]. In this case, the Xuan et al. [30] model has the smallest error at 4.377%. It correctly predicted an increase in thermal conductivity with respect to the volume fraction; however, it overestimated the decrease due to increasing the aggregate size between 5 nm and 30 nm. The Prasher et al. [29] model initially underestimates the thermal conductivity, but corrects for it by predicting a significant increase with the volume fraction. Lastly, both the Feng et al. [31] and Wang et al. [32] models underpredict the thermal conductivity in general, but more accurately convey its increase as the volume fraction increases. Note that since aggregate size changed with the volume fraction according to an unknown correlation, the actual curve between data points of the Prasher et al. [29] and Xuan et al. [30] models could not be conveyed. The Feng et al. [31] and Wang et al. [32] curves are still accurate since the models are independent of $r_{a}$.

Table 4 shows the level of accuracy of each aggregation model for each set of experimental data used. Due to the scarcity of aggregation data provided in combination with the thermal conductivity data in the existing literature, only five datasets were used to analyze the relative accuracy of each aggregation model. It can be seen that the Prasher et al. [29] model gave the smallest error observed when compared against the data for the CuO–EG nanofluid. At the same time, the model was inconsistent relative to the others and disagreed with the Al_2_O_3_ datasets. Meanwhile, both the Xuan et al. [30] and Feng et al. [31] models were the most accurate for two datasets each. Of the two, the Feng et al. [31] model could be considered better because its range of error is much smaller than that observed for the Xuan et al. [30] model. The Wang et al. [32] is also a stable model and was only slightly less accurate on average than the Feng et al. [31] model.

### 2.5. Molecular Dynamics Simulations

Molecular dynamics simulations attempt to model the thermal conductivity of a nanofluid by calculating all the interactions between all particles within the nanofluid. These simulations can be used to understand molecular interactions, that would not be possible to measure experimentally, by solving the classical equations of motion at the atomic scale [45]. Evans et al. [33] used molecular dynamics simulations to predict the thermal conductivity of nanofluids. They considered only stationary fluids, for which they discovered that Brownian motion had a negligible impact on the thermal conductivity. In addition, particles were not found to cluster in aggregates. Thus, it was determined the enhancement predicted by EMT most accurately described the results. In their simulation, they assumed that the particle density was 450% the density of the base fluid. Eight nanoparticles suspended in 50,000 molecules of base fluid were simulated. In other words, this simulated a fluid made up of approximately 3.3% nanoparticles by volume. Three different types of simulations were ran based on different interaction strengths of the base fluid with the particles. Specifically, the simulations had no wetting, weak wetting, or wetting. Based on their work, the Evans et al. [33] model is given as:

(39) $$k_{nf}=k_{bf}\left(1+3\phi \frac{\epsilon -1}{\epsilon +2}\right),\;$$

where $\epsilon =r_{p}/h$ is the ratio of the particle radius $r_{p}$ to the equivalent matrix thickness h. This ratio is a measure of the level of interfacial resistance between the base fluid and nanoparticles. A constant ratio of 2.9 was assumed for analysis, as this was the median of the boundaries used to produce the model. Additionally, particle radii and makeup were constant for each independent dataset analyzed, so the ratio was also constant within datasets. The Evans et al. [33] model suggests a direct correlation between particle size and the thermal conductivity. Specifically, the thermal conductivity would increase due to a decrease in the thermal resistance at the interfacial boundary. Compared to other molecular dynamics models [34,35], the Evans et al. [33] model is relatively straightforward. Although, it requires the use of the equivalent matrix thickness, which generally must be assumed. Additionally, the model is not capable of accounting for the effects of Brownian motion or aggregation.

Vladkov and Barrat [34] more rigorously considered the effects of interfacial resistance on thermal conductivity enhancement. They ran a simulation to determine the heat flux through a system consisting of a particle with a radius of 1.5 nm in a base fluid with a side length of 10 nm. The determined heat flux was then used to solve for the thermal conductivity. The Vladkov and Barrat [34] model is given as:

(40) $$k_{nf}=k_{bf}\left(\frac{\left(\frac{k_{p}}{k_{bf}}\left(1+2\Omega \right)+2\right)+2\phi \left(\frac{k_{p}}{k_{bf}}\left(1-\Omega \right)-1\right)}{\left(\frac{k_{p}}{k_{bf}}\left(1+2\Omega \right)+2\right)-\phi \left(\frac{k_{p}}{k_{bf}}\left(1-\Omega \right)-1\right)}\right),$$

where $\Omega =R_{k}k_{bf}/r_{p}$ is the ratio of the equivalent thermal thickness to the particle radius. For this model, the equivalent interfacial nanolayer’s thermal thickness was assumed to be unity, simplifying calculations to $\Omega =1/r_{p}$. This assumption was made due to a lack of data regarding interfacial conductance. The interfacial conductance is the inverse of the Kapitza resistance, $R_{k}$. By assuming that the interfacial conductance is equal to the conductance of the base fluid, the thermal thickness $R_{k}k_{bf}$ becomes equal to one. When this assumption is made, the model predicts the thermal conductivity to be a strong function of particle size, with the thermal conductivity increasing as the particle radius decreases. Vladkov and Barrat [34] were able to rigorously consider the effects of the thermal interface between the nanoparticle and surrounding base fluid. This would be an advantage; however, the resulting characteristics are difficult to measure. Thus, without more information on parameters such as the equivalent thermal thickness, the model is difficult to apply. Additionally, the effects of Brownian motion and aggregation were not considered.

Lin et al. [35] created molecular dynamics simulations using ethylene-glycol-based Cu nanofluid to calculate its thermal conductivity. They sought to consider the effects of the nanolayer on the thermal conductivity. The simulation produced by Lin et al. [35] consisted of 3% Cu by volume suspended in 1200 ethylene glycol molecules. The model produced from the simulation combines aspects of both the Maxwell [14] EMT and the Yu and Choi [27] nanolayer models. The Lin et al. [35] model looks at a three-part nanofluid consisting of the nanoparticles, the base fluid, and the well-ordered nanolayer around the particle. The model was derived from the spherical form of the heat conduction equation and is given as:

(41) $$k_{nf}=k_{bf}+\frac{\left(k_{p}-k_{bf}\right)\left(\frac{\left(1-\zeta_{1}\right)\left(1-\zeta_{2}\right)}{1+2\rho_{nf}\zeta_{1}\zeta_{2}}\right)\phi_{p}+\left(k_{nl}-k_{bf}\right)\left(\frac{1-\zeta_{1}}{1+2\rho_{nf}\zeta_{1}\zeta_{2}}\right)\left(\phi_{pl}-\phi_{p}\right)}{\left(\frac{\left(1-\zeta_{1}\right)\left(1-\zeta_{2}\right)}{1+2\rho_{nf}\zeta_{1}\zeta_{2}}\right)\phi_{p}+\left(\frac{1-\zeta_{1}}{1+2\rho_{nf}\zeta_{1}\zeta_{2}}\right)\left(\phi_{pl}-\phi_{p}\right)+\left(1-\phi_{pl}\right)},\;$$

(42) $$\zeta_{1}=\frac{k_{p}-k_{nl}}{k_{p}+2k_{nl}},$$

(43) $$\zeta_{2}=\frac{k_{nl}-k_{bf}}{k_{p}+2k_{nl}}.$$

$\zeta_{1}$ and $\zeta_{2}$ dimensionlessly relate the nanolayer’s thermal conductivity to the particle and base fluid’s thermal conductivities, respectively, and $\phi_{pl}$ is the volume fraction of the nanoparticle added to the volume fraction of the nanolayer. The advantage of the model by Lin et al. [35] is that it considers the nanolayer, unlike the remaining molecular dynamics models [33,34,36]. Again, this comes with the downside that the nanolayer is not yet understood. Consequently, significant assumptions are required for its application.

Mirmohammadi et al. [36] created a molecular dynamics simulation using Ag nanoparticles suspended in water. This simulation intended to look at the effects of the particle shape and concentration as well as nanofluid temperature to get a better understanding of how each parameter affected thermal conductivity enhancement. The particles used in the simulation included spherical, cylindrical, and rectangular prismatic nanoparticles. Concentrations between 0.14% and 1.4% by volume were investigated at temperatures between 7 °C to 62 °C. Through this simulation, Mirmohammadi et al. [36] discovered that the ratio of the nanoparticle surface area to volume was the most important shape parameter. Additionally, they verified prior findings that showed increased concentration and temperature were correlated with an increase in the nanofluid thermal conductivity. The simulation results were verified by experimental data using similar Ag–water nanofluids. The Mirmohammadi et al. [36] model is given as:

(44) $$k_{nf}=k_{bf}\left(1+9.89{\left(\frac{T}{300}\right)}^{4.75}{\left(\phi \right)}^{0.65}{\left(0.30855\frac{A_{s}}{V}\right)}^{0.23}\right),$$

where $A_{s}/V$ is the ratio of surface area to the volume of the nanoparticle. The model used was determined by comparing the simulation data with experimental data and nondimensionalizing each parameter. It suggests an asymptotic relationship between the surface area to volume ratio and the thermal conductivity. In other words, as the ratio increases, the thermal conductivity will approach a constant value determined by the other characteristics. Of all the molecular dynamics models investigated [33,34,35], Mirmohammadi et al. [36] formulated the most straightforward model. It only requires knowledge of the concentration, temperature, and ratio of the surface area to volume of the particles. However, it does have a disadvantage, considering that it does not account for the effect of the particle material.

**Figure 10:**
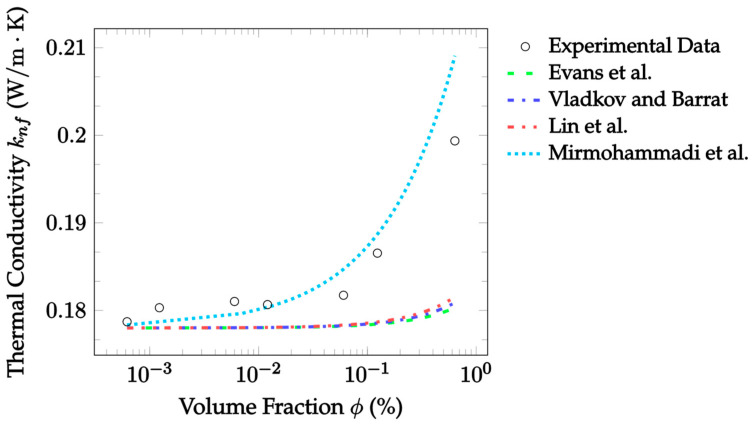
Molecular dynamics simulation predictions compared to experimental data for CuO nanoparticles in ethanol at T=300 K, for volume fractions between $6.18\times 10^{-4}$ and 0.637%. Experimental data [78], Evans et al. [33], Vladkov and Barrat [34], Lin et al. [35], Mirmohammadi et al. [36].

Figure 10 below shows nanofluids thermal conductivity as a function of volume fraction for CuO nanoparticles dispersed in water at room temperature for very low volume fractions. The Mirmohammadi et al. [36] model proved the most accurate, with an MAE of 1.409%. As can be seen in the graph, the model predicted the large increase in thermal conductivity from volume fractions of 0.1–0.64% with a higher accuracy compared to the other models examined. While all models did predict an increase in thermal conductivity with an increasing volume fraction, the remaining three models assumed that the change at the higher volume fractions found in this dataset would be very small compared to the actual experimental findings, ultimately underpredicting the thermal conductivity at higher volume fractions. Despite this, all four models examined against this dataset predicted the thermal conductivity relatively well, with mean average errors of less than 3%.

**Figure 11:**
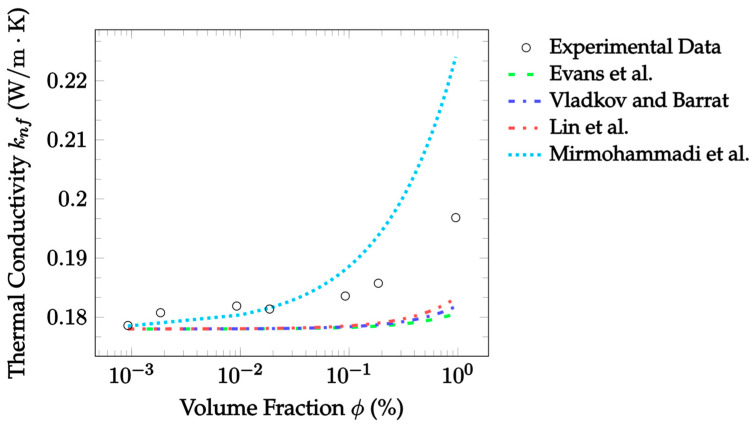
Molecular dynamics simulation predictions compared with experimental data for TiO_2_ in ethanol for T=300 K, volume fractions between $9.09\times 10^{-4}$ and 0.957%: Experimental data [78], Evans et al. [33], Vladkov and Barrat [34], Lin et al. [35], Mirmohammadi et al. [36].

Figure 11 displays the change in thermal conductivity with volume fraction for TiO_2_ nanoparticles dispersed in ethanol at room temperature. Similar to Figure 10, this experimental data is for low-concentration nanofluids, with concentrations ranging from $9.09\times 10^{-4}$ to 0.957%. This time, the Lin et al. [35] model predicted the thermal conductivity the most accurately, with an MAE of 2.724%. However, this was not significantly better than the Evans et al. [33] or Vladkov and Barrat [34] models, which had mean average errors of 2.979% and 2.863%, respectively. Even the Mirmohammadi et al. [36] model, which appears to be the least accurate compared to the remaining models at volume fractions greater than roughly 0.2%, only had a slightly higher MAE of 3.249%. All four models made similar predictions as in Figure 10, but the thermal conductivity in this case was a weaker function of the volume fraction, resulting in a lower thermal conductivity at the higher volume fractions. This caused the Mirmohammadi et al. [36] model’s error to be higher for high volume fractions, resulting in the highest MAE for this dataset.

**Figure 12:**
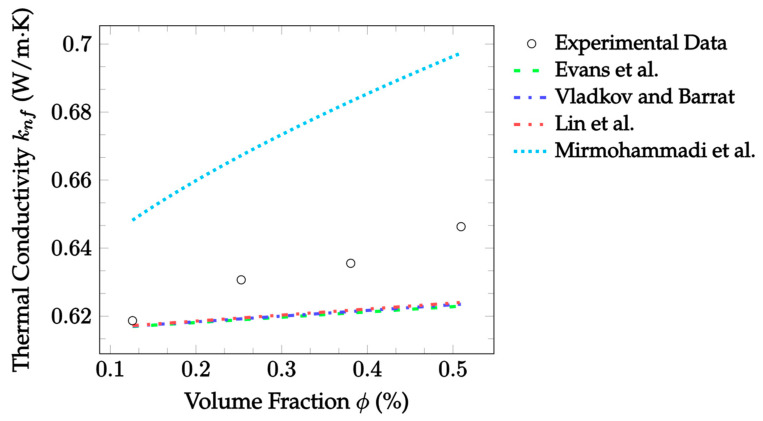
Molecular dynamics simulations compared with experimental data for 100 nm diameter Al_2_O_3_ nanoparticles in water at T = 30 °C, with volume fractions between 0.126 and 0.5%: Experimental data [79], Evans et al. [33], Vladkov and Barrat [34], Lin et al. [35], Mirmohammadi et al. [36].

In Figure 12, experimental data for the thermal conductivity of Al_2_O_3_-water at 30 °C as a function of volume fraction is shown. Compared to the previous graphs, a much narrower range of concentrations is examined, only ranging from 0.126% to 0.5%. Similar to Figure 11, the Lin et al. [35] model had the lowest MAE at 1.906%, with the Evans et al. [33] and Vladkov and Barrat [34] models following closely behind with mean average errors of 2.013% and 1.957%, respectively, all within nearly 0.1% of each other. Unlike the other models, the Mirmohammadi et al. [36] model has a significantly higher error than the other models treated, with an MAE of 6.490%. This is due to the model overestimating the effect of increasing concentration on the thermal conductivity. Therefore, overestimation exhibited by the Mirmohammadi et al. [36] model is more severe than the underestimation of the remaining three predictions [33,34,35]. Regarding the consistently larger errors, this is due to the concentrations in this case being relatively higher than the cases in Figure 10 and Figure 11.

Table 5 below shows the mean average error for each model examined against ten different datasets from three different experiments. Generally, all four models tended to perform best at lower temperatures, with errors increasing as the temperature was increased. Additionally, it can be seen that most models appeared to get more accurate as the particle diameter increased. The exception is the Mirmohammadi et al. [36] model, which showed no clear correlation between accuracy and particle diameter. The Mirmohammadi et al. [36] model performed significantly better than the other models when considering CuO–ethanol and TiO_2_–ethanol nanofluid at higher temperatures. For each of these datasets, the volume fraction ranges were relatively low with maximum values below 1.0%. As the volume fraction increased further, the model began to significantly overestimate the thermal conductivity, leading to the very high errors observed in the last two data sets. The Evans et al. [33], Vladkov and Barrat [34], and Lin et al. [35] models made nearly identical predictions in all cases, as seen in both the above Figures and the mean average errors in the table below.

### 2.6. Thermal Conductivity Models for Hybrid Nanofluids

In this section, a brief discussion on the topic of hybrid nanofluids is provided, and then the level of accuracy of various general hybrid nanofluid models are examined using experimental data. It was observed that there is a lack of theoretical models for hybrid nanofluids with a broad validity range. The current theoretical models indicated that there is a need for improvement in the level of accuracy of existing general hybrid nanofluid models.

A hybrid nanofluid is defined as a colloidal suspension of dissimilar nanoparticles. These particles can either be distinct or combined into composite nanoparticles. The reason behind the sudden interest in hybrid nanofluids could have to do with the reported finding that they may yield a better thermal performance than mono nanofluids [37,46,47]. It is believed that this can be attributed to greater control over their properties (i.e., the deficiencies of the first nanoparticle type could be covered by the addition of a second nanoparticle type) as well as the synergistic effect [56]. Generally, the synergistic effect in nanofluid research refers to an additional improvement from nanoparticles working in combination. This would be in addition to the individual particles’ standalone effects. Obviously, hybrid and ternary hybrid nanofluids have a significantly higher number of parameters that need to be considered. When engineering a nanofluid or developing a model to predict its properties, every characteristic could be important. Not only is there a set of characteristics for each particle, but the mixture ratio is also an important factor. Therefore, the engineering of hybrid nanofluids is necessary.

It was observed that many examples of thermal conductivity models for a given hybrid nanofluid mixture were formulated purely through empirical correlations [9,80,81,82,83,84,85,86]. Common input parameters included the net volume fraction of all nanoparticle types as well as the temperature. However, only a very small fraction of these models was found to be applicable to a general hybrid nanofluid, considering different particle types, base fluids, and nanoparticle mixture ratios. This is likely due to the large number of parameters to be accounted for in hybrid nanofluids. In the context of this paper, the validity ranges of the existing empirical models were much too narrow to compare their levels of accuracy. Thus, the more general hybrid nanofluid models were selected for treatment. Additionally, the empirical models do not have the scope necessary for the engineering of hybrid nanofluids.

Chamkha et al. [37] modified the Maxwell [14] EMT model to allow its application to hybrid nanofluids. The methodology used was to assume that the particle thermal conductivity of the single nanoparticle type could be replaced with an equivalent particle thermal conductivity, taking into account both material types. This was completed by using an average of the constituent particles’ thermal conductivities, weighted with respect to their volume fractions. Then, the hybrid nanofluid concentration was considered as the sum of the constituent particles’ concentrations. Given that the model is based on the Maxwell [14] model, it could be considered a hybrid EMT model. It also uses the same assumptions as the Maxwell [14] model. Specifically, inter-particle interaction does not occur, and the nanoparticles are spherical. The Chamkha et al. [37] model for hybrid nanofluids is displayed in Equation (45):

(45) $$k_{hnf}=k_{bf}\left[\frac{\frac{\phi_{1}k_{p,1}+\phi_{2}k_{p,2}}{\phi_{hnf}}+2k_{bf}+2\left(\phi_{1}k_{p,1}+\phi_{2}k_{p,2}\right)-2k_{bf}\phi_{hnf}}{\frac{\phi_{1}k_{p,1}+\phi_{2}k_{p,2}}{\phi_{hnf}}+2k_{bf}+\left(\phi_{1}k_{p,1}+\phi_{2}k_{p,2}\right)-k_{bf}\phi_{hnf}}\right],$$

where $\phi_{1}$, $k_{p,1}$, $\phi_{2}$, and $k_{p,2}$ are the individual concentrations and particle thermal conductivities of the nanoparticle constituents, and $\phi_{hnf}=\phi_{1}+\phi_{2}$ is the nanoparticle concentration of the hybrid nanofluid. Again, Chamkha et al. [37] predicted the thermal conductivity based on an effective, statistically averaged particle that does not truly exist. Therefore, it does not realistically model a nanofluid with two distinct nanoparticles. This may be seen as a disadvantage.

Another general model was developed by Devi and Devi [38], who instead considered the Hamilton and Crosser [15] model as their starting point. A different procedure was used in this case, where a hybrid nanofluid was considered to be a mono nanofluid, with a separate nanofluid as a base fluid. The advantage of considering the Hamilton and Crosser [15] model over the Maxwell [14] model is that it takes into account the nanoparticle shape as an additional mechanism of enhancement. As a result, the model only assumes that inter-particle interactions do not occur. It does, however, additionally assume that the effects of the dissimilar particles on thermal conductivity are purely multiplicative. The resulting model is displayed in Equation (46) below:

(46) $$k_{hnf}=k_{bf}\left[\frac{k_{p,1}+\left(n_{1}-1\right)k_{bf}-\left(n_{1}-1\right)\phi_{1}\left(k_{bf}-k_{p,1}\right)}{k_{p,1}+\left(n_{1}-1\right)k_{bf}+\phi_{1}\left(k_{bf}-k_{p,1}\right)}\right]\left[\frac{k_{p,2}+\left(n_{2}-1\right)k_{bf}-\left(n_{2}-1\right)\phi_{2}\left(k_{bf}-k_{p,2}\right)}{k_{p,2}+\left(n_{2}-1\right)k_{bf}+\phi_{2}\left(k_{bf}-k_{p,2}\right)}\right],$$

where $n_{1}$ and $n_{2}$ are the shape factors of their respective particle constituents. While the Chamkha et al. [37] model only considered a mono nanofluid with effective nanoparticles, Devi and Devi [38] accurately modeled both particles in suspension. The model also considers the nanoparticle shape, which cannot be said for the other hybrid nanofluid models [37,39].

At last, Chougule and Sahu [39] derived a hybrid nanofluid thermal conductivity model using the methodology of Kumar et al. [24], whose Brownian model was investigated in Section 2.2. A one-dimensional heat transfer network was used, where there exist two parallel paths of heat transfer, one through the nanoparticles and one through the base fluid. In this model, it was assumed that the base fluid and all nanoparticles were in local thermal equilibrium. The heat transfer areas were taken to be the combined surface areas of the nanoparticle constituents and base fluid particles, where it was additionally assumed that all molecules were spherical. Unlike the previous two models discussed, Chougule and Sahu [39] take into account the enhancement due to particle size. The model is also derived directly from a heat transfer model considering Brownian motion, while Chamkha et al. [37] incorporate hybrid nanofluids in a more statistical fashion. Finally, the model derived by Chougule and Sahu [39] is presented below:

(47) $$k_{hnf}=k_{bf}\left[1+\frac{k_{p,1}\phi_{1}r_{bf}}{k_{bf}\left(1-\phi_{hnf}\right)r_{p,1}}+\frac{k_{p,2}\phi_{2}r_{bf}}{k_{bf}\left(1-\phi_{hnf}\right)r_{p,2}}\right],$$

where $r_{p,1}$ and $r_{p,2}$ are the radii of the respective nanoparticle constituents. Again, this was the only hybrid nanofluid model of those discussed [37,38] to consider Brownian motion, which is a significant advantage. However, it does require knowledge of the base fluid’s particle size. Fortunately, this is a known parameter for many of the commonly used base fluids.

**Figure 13:**
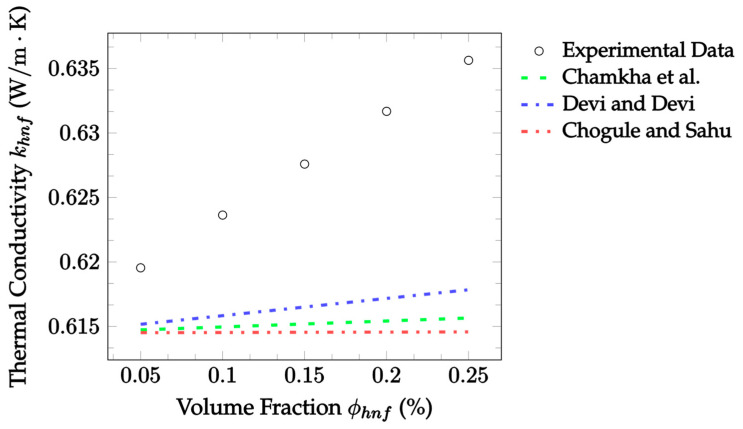
Experimental findings for 50:50 TiO_2_–CaCO_3_/Water hybrid nanofluid compared with theoretical predictions by general hybrid nanofluid models: Experimental Data [87], Chamkha et al. [37], Devi and Devi [38], Chougule and Sahu [39].

**Figure 14:**
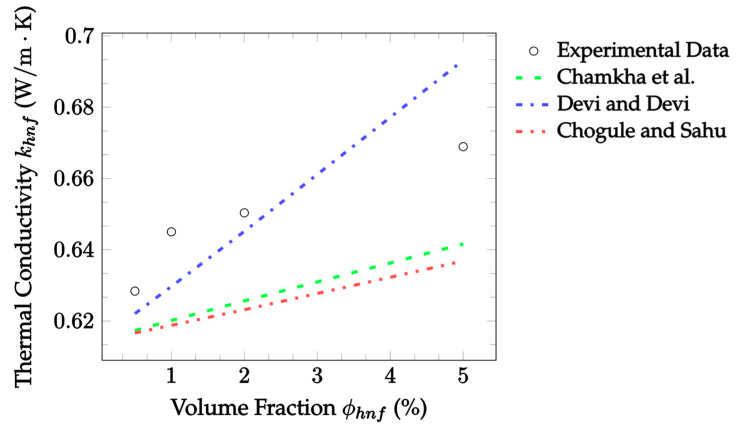
Experimental findings for 10:1 TiO_2_–Ag/Water hybrid nanofluid compared with more theoretical predictions: Experimental Data [88], Chamkha et al. [37], Devi and Devi [38], Chougule and Sahu [39].

Figure 13 below displays the theoretical predictions for the hybrid nanofluid thermal conductivity against existing experimental data. While all MAEs calculated were around 2%, it is clear that these models underestimate the thermal conductivity in general. The Devi and Devi [38] model performed best with an MAE of 1.763%, whereas the Chougule and Sahu [39] model struggled to capture the effects of the volume fraction on thermal conductivity enhancement. This underestimation in all models was found to be a reoccurring issue in the remaining datasets except for one, which is shown in Figure 14 for the sake of completeness. Again, the Devi and Devi [38] model has the lowest MAE with 1.952%. However, it could be argued that the Chamkha et al. [37] and Chougule and Sahu [39] models perform better in this case, as they correctly predict the rate of enhancement with changes in concentration past ϕ=1%. The Devi and Devi [38] model only shows a lower error because of the sharp, nonlinear improvement in the thermal conductivity at smaller volume fractions of ϕ≤1%.

In Table 6 below, the MAE between each model and dataset analyzed is given. Recent data were gathered from nine different sources, and had varying particle ratios, concentrations, sizes, temperatures, particle materials, and shapes. An examination of the data reveals that the Devi and Devi [38] consistently performed better than the other two models, having the lowest MAE in eight of the nine datasets analyzed. Given that at a foundational level, the Hamilton and Crosser [15] and Maxwell [14] models are equivalent for spherical particles, this suggests its good performance has to do with how hybrid nanoparticles were implemented. It is recalled that Chamkha et al. [37] used effective material properties found through statistical averaging, whereas Devi and Devi [38] modeled the base fluid as a separate mono nanofluid. The data suggest that the latter is a better approach. The Chamkha et al. [37] model performed better than the Chougule and Sahu [39] model in six of the nine total cases; however, the differences in MAE were most often marginal. As a result, it is concluded that the models perform similarly.

## 3. Comprehensive Thermal Conductivity Model for General *N* Hybrid Nanofluids

The focus of this paper is to develop a new model that can be applied to a large range of nanofluids. The level of accuracy of the model is examined with independent experimental data and a good agreement was observed. There exists many models that can be applied to a very specific hybrid nanofluid. Most commonly, the base fluids, particle types, particle mixture ratios, and even, on occasion, the temperatures, are all fixed parameters that these models do not account for. The few existing models that can be applied to a more general hybrid nanofluid were analyzed in Section 2.6. However, they only apply to nanofluids with two distinct nanoparticle types. Then, it is the aim of this paper to confront this issue. Firstly, a method to convert a mono nanofluid to a hybrid nanofluid model is developed. These models will all be applicable to not only general hybrid nanofluids, but hybrid nanofluids with any number of distinct particles. Secondly, this method will be utilized to create a new model that maintains a sufficiently high level of accuracy over the large domain.

After examining many different mono nanofluid models, a pattern is observed between most of them. Namely, many follow the structure of predicting the nanofluid thermal conductivity as a multiplication between the base fluid’s thermal conductivity and some enhancement term λ, where λ is a function of the nanofluid’s properties. In other words, it can be said that $k_{nf}=k_{bf}\lambda_{1}$. Now, let us say that $k_{hnf}=k_{nf}\lambda_{2}$, where $\lambda_{2}$ is the enhancement coefficient due to the second nanoparticle loading. However, $k_{nf}$ was already expressed in terms of the base fluid’s thermal conductivity and $\lambda_{1}$, the enhancement due to the first nanoparticle’s loading. So, combining these two equations:

(48) $$k_{hnf}=k_{bf}\lambda_{1}\lambda_{2}.$$

This pattern of logic can be continued for N nanoparticle loadings; thus, a general framework is proposed for an N hybrid nanofluid model based on a mono nanofluid model:

(49) $$k_{Nhnf}=k_{bf}\overset{N}{\underset{i=1}{\prod }}\lambda_{i}.$$

Here, it is assumed that the impact of interactions between distinct nanoparticles on the thermal conductivity is negligible. In other words, it assumes that there is no synergistic effect taking place between nanoparticle types.

Assuming the $\lambda_{i}$ are all mutually independent of one another, this framework will predict the same value regardless of the order of operations during nanofluid synthesis. This is a direct consequence of the commutativity of multiplication. Changing the order of nanoparticle introduction merely changes the order of multiplication of the enhancement terms, and thus does not change the result. Now, an expression for $\lambda_{i}$ must be found. The Devi and Devi [38] model for N nanoparticle loadings can be derived if the Hamilton and Crosser [15] model is chosen. However, in this paper, a different model is used. The model with the highest level of accuracy according to the performance analysis in Section 2 is the Patel et al. [23] Brownian model. Therefore, it is selected as the base mono nanofluid model. So, according to Patel et al. [23], the following expression should be used for $\lambda_{i}$:

(50) $$\lambda_{i}=1+0.135{\left(\frac{k_{p,i}}{k_{f,i-1}}\right)}^{0.273}\phi_{i}^{0.467}{\left(\frac{T}{20}\right)}^{0.547}{\left(\frac{100}{d_{p,i}}\right)}^{0.234},$$

where $k_{f,i-1}$ is the thermal conductivity of the base fluid for the ith hybrid nanofluid ($k_{f,0}=k_{bf}$). Equivalently, $k_{f,i-1}$ is the thermal conductivity for the (i−1)th hybrid nanofluid, and $k_{p,i}$, $\phi_{i}$, and $d_{p,i}$ are all properties of the ith nanoparticle. However, because $\lambda_{i}$ is now dependent on the base fluid properties of each hybrid nanofluid, the previously made assumption that all $\lambda_{i}$ terms are mutually independent is violated. This means that, without correction, the order of operations can alter the predicted value. While this change was found to be small when computing values for the given data (maximum order of $10^{-6}$) this issue can be easily avoided if the product of the enhancement terms is assumed to purely be a function of the original base fluid, instead of all N base fluids. Mathematically speaking, $k_{f,i-1}=k_{bf}$ for all whole values of i. Substituting these equations into the general framework in Equation (49), a new correlation is proposed for general N hybrid nanofluids:

(51) $$k_{Nhnf}=k_{bf}\overset{N}{\underset{i=1}{\prod }}\left(1+0.135{\left(\frac{k_{p,i}}{k_{bf}}\right)}^{0.273}\phi_{i}^{0.467}{\left(\frac{T}{20}\right)}^{0.547}{\left(\frac{100}{d_{p,i}}\right)}^{0.234}\right).$$

Here, T is expressed in °C, and $d_{p}$ in nm. Additionally, because the work of Patel et al. [23] is being utilized, it is assumed that all particles are spherical. The model also has a validity range of $10\;\mathrm{nm}\leq d_{p}\leq 150\;\mathrm{nm}$, $20\;W/m\cdot K\leq k_{p}\leq 400\;W/m\cdot K$, $0.1\;W/m\cdot K\leq k_{bf}\leq 0.7\;W/m\cdot K$, $0.1\%\leq \phi_{Nhnf}\leq 3\%$, and 20 °C≤T≤50 °C, where:

(52) $$\phi_{Nhnf}=\overset{N}{\underset{i=1}{\sum }}\phi_{i}.$$

**Figure 15:**
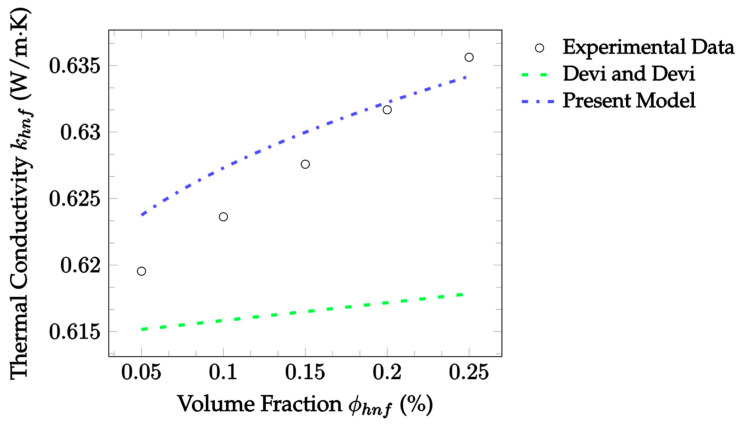
Present model predictions vs. best existing model for experimental findings of 50:50 TiO_2_–CaCO_3_/Water hybrid nanofluid: Experimental Data [87], Devi and Devi [38].

The performance of the new model compared with the best performing hybrid nanofluid model (Devi and Devi [38]) is illustrated in Figure 15 and Table 7. In Figure 15, the present model is shown to not exhibit the same underestimation issues that the other xhybrid nanofluid models showed. This may have to do with the inclusion of Brownian motion as a mechanism of thermal conductivity enhancement, since the Devi and Devi [38] model utilized EMT fundamentally. In this case, the MAE for Devi and Devi [38] was 1.763%, while the present model had an MAE of 0.393%.

In Table 7 below, the MAE between each dataset used in Section 2.6 and the Devi and Devi [38] and present models are displayed. For eight of the nine datasets, the present model had a lower MAE than the existing model. In fact, for many of the datasets, the value was significantly lower. The only exception to this is the dataset using 10:1 TiO_2_–Ag/Water nanofluid. This may have to do with the fact that Brownian motion does not provide much enhancement for this specific nanofluid.

**Figure 16:**
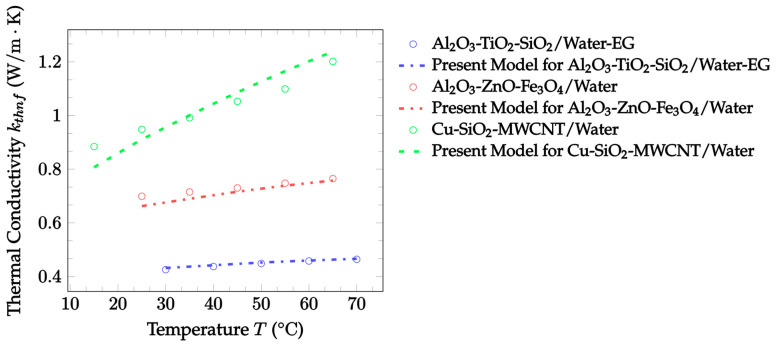
Present model predictions vs. experimental data for ternary hybrid nanofluid. For this figure, predictions at temperature values other than those for experimental data are not accurate: Al_2_O_3_-TiO_2_-SiO_2_/Water–EG [95], Al_2_O_3_-ZnO-Fe_3_O_4_/Water [96], Cu-SiO_2_-MWCNT/Water [97].

Here, it is reminded that the present model can also predict the thermal conductivity for nanofluids with more particle types. The next nanofluid in line is the ternary hybrid nanofluid, for which N=3. Figure 16 shows present model predictions for various ternary hybrid nanofluids. The first set was for 1:1:1 Al_2_O_3_–TiO_2_–SiO_2_/60:40 Water–EG nanofluid, with a volume fraction of 0.1% [95]. The model is in excellent agreement with the data, having an MAE of 0.831%. The following set was for 1:1:1 Al_2_O_3_–ZnO–Fe_3_O_4_/Water nanofluid with a volume fraction of 1% [96]. While the model had slightly less accurate predictions in this case, it was still in good agreement with an MAE of 2.580%. Finally, the last dataset was for 1:1:1, Cu–SiO_2_–MWCNT/Water with a volume fraction of 3% [97]. Again, the model performed slightly worse than the previous two datasets. However, it still agreed with experimental results, having an MAE of 4.320%. It is believed that the higher MAE is due to the significantly higher volume fraction, as well as the use of MWCNT nanoparticles which were not spherical (n=11.4). The good agreement with the experimental data could mean the synergistic effect is negligible in these nanofluids.

The effectiveness of nanofluids in thermal management depends on the nanofluid characteristics, which can be modified by adding a second type of nanoparticle. For instance, the deficiencies of the first nanoparticle type could be covered by the second nanoparticle type. This gives hybrid nanofluids an advantage over mono nanofluids in general. In addition, more nanoparticle types could be added if further modifications are necessary. A model of the form presented in this paper has a significant impact on the future development of hybrid nanofluids. For the first time, it permits a direct analytical comparison between hybrid nanofluids with different numbers of distinct nanoparticles. Therefore, it can be used to engineer hybrid nanofluids when the number of distinct nanoparticles is a factor of consideration. Most often, this is the case, as it is rarely a fixed parameter in preliminary design. Hybrid nanofluids themselves are important because the number of modifiable parameters is significantly higher than that found in mono nanofluids. Consequently, there exists a stronger control over their effective characteristics. This is beneficial in many real-world applications, where compromises between these parameters are necessary [98]. For instance, hybrid nanofluids are commonly used in solar collectors, as the absorption range and thermal conductivity of the fluid can be tuned to optimize energy efficiency [99]. Thus, researchers will only put greater emphasis on the investigation of hybrid nanofluids with time.

## 4. Nanofluid Viscosity Models

In this section, various models describing the viscosity of nanofluids are presented, and their levels of accuracy are examined by existing experimental data. Viscosity is a key property in describing nanofluids due to its implications in energy applications. Nanofluid viscosity is a property dependent on many factors such as temperature, base liquid, and the nanoparticle concentration and characteristics. These relationships have been vastly studied and confirmed through a large quantity of experiments. The most widely agreed upon result is the effect of temperature. As the temperature of a nanofluid increases, the viscosity is found to decrease. This relationship is possibly due to the weakening of inter-molecular forces and decreased stability [100]. This predicted result has been verified experimentally by many existing studies. One such example is the work of Sundar et al. [18], who studied Fe_3_O_4_ nanoparticles in water between 20 °C to 60 °C. It was found that the dynamic viscosity decreased by 46.2% over the given temperature range. Another instance is the experiment carried out by Sharma et al. [101]. They found that the dynamic viscosity of a CuO–water nanofluid decreased by 53.7% over a range of 30 °C to 80 °C. Nanofluid viscosity is also dependent on the base liquid used. This could be because the nanofluid viscosity is directly proportional to that of the base fluid [100]. Additionally, nanoparticle concentration has a significant effect on the viscosity. As more particles are loaded into the fluid, the viscosity significantly increases. This may be due to the high surface area to volume ratio of the nanoparticles, resulting in greater resistance to flows [102]. This was observed experimentally by Sharma et al. [101], who studied CuO–water nanofluids with concentrations of 0.02% and 0.1% and the base liquid. In addition, Ahammed et al. [103] reached the same conclusion with graphene–water nanofluids at volume fractions of 0.05%, 0.1% and 0.15%. Nanoparticle size is also commonly examined. Size and nanofluid viscosity are typically reported to have an inverse relationship, where viscosity will increase with a decreasing nanoparticle diameter. This could be due to the increased number of particle agglomerates formed with smaller-diameter particles [100]. Zhang et al. [104] verified this effect with an experimental study on 15, 30, and 80 nm SiO_2_ particles in water. Specifically, the viscosity enhancement was deemed to be 9.2%, 8.3%, and 6.1%, respectively, as the diameter increased. The same effect for SiC–water nanofluids was studied by Timofeeva et al. [105]. They used more particle sizes of 16, 29, 66, and 90 nm. At a given temperature and volume concentration, the 16 nm SiC–water nanofluid experienced the greatest viscosity, and the 90 nm the least. With all these effects in mind, researchers have developed many models with the objective of predicting nanofluid viscosity. A brief summary on each of these models can be seen in Appendix B, Table A3.

One of the earliest models describing the viscosity of a nanofluid was presented by Brinkman [48] in 1951. Brinkman [48] proposed a simple model where the viscosity of a solution is a function of the concentration of nanoparticles in a nanofluid. It was determined by following a method similar to Onsager [106] in his theory on the dielectric constant, and by modifying Einstein’s model. The model is given as the following:

(53) $$\mu_{nf}=\frac{\mu_{bf}}{{\left(1-\phi \right)}^{2.5}},$$

where $\mu_{nf}$ is the viscosity of the nanofluid, and $\mu_{bf}$ is the base fluid’s viscosity. The Brinkman [48] model was derived by modifying Einstein’s well-known model describing the viscosity increase as an effect of adding a singular particle to a pure solvent. This model operates under the assumptions that the nanoparticles are spherical. In addition, it assumes the Einstein factor and base liquid viscosity apply when many particles are in suspension. The second assumption considers the nanofluid as a pure solvent with the same viscosity, which in the above expression could be exact if the nanoparticles existing in the nanofluid are much smaller than the one being added. The Brinkman [48] model neglects to consider any effect other than that of the concentration on the nanofluid viscosity. Factors that other nanofluid viscosity models include [49,50,51,52] such as particle size and shape, density, and temperature, were not considered.

Meybodi et al. [49] also developed a nanofluid viscosity model and utilized inputs of temperature, nanoparticle size, volume fraction, and base fluid viscosity. Their aim was to accurately predict the viscosity in high-temperature, concentration, and viscosity nanofluids. From previously conducted experimental investigations on water-based nanofluids with Al_2_O_3_, TiO_2_, SiO_2_, and CuO nanoparticles, the following model was developed:

(54) $$\mu_{nf}=\mu_{bf}\times \frac{133.5406-343.8241\exp\left(\phi /d_{p}\right)+290.1180\exp{\left(\phi /d_{p}\right)}^{2}-78.9931\exp{\left(\phi /d_{p}\right)}^{3}}{0.9116+32.3301\left(\frac{\ln\left(d_{p}\right)}{T}\right)-11.7325\left(\frac{\ln{\left(d_{p}\right)}^{2}}{T}\right)}.$$

In this model, $d_{p}$ is expressed in nm, and T is expressed in K. Coefficients were determined using nonlinear regression driven by minimizing the mean square error. The primary assumption is that the base fluid is water.

Masoumi et al. [50] introduced another viscosity model that considers the Brownian motion of the nanoparticles. It presents the viscosity as a function of nanoparticle size, density, volume concentration, the temperature, density, and the base fluid’s physical properties. The effective viscosity of the nanofluid $\mu_{nf}$ is represented by the following equation:

(55) $$\mu_{nf}=\mu_{bf}+\frac{\rho_{p}u_{B}d_{p}^{2}}{72C\xi },$$

where C is a correction factor, $\rho_{p}$ is the nanoparticle density, and ξ is the distance between nanoparticles, assuming a homogeneous distribution. In this expression, the particle’s initial kinetic energy is assumed to be equal to the work done by surface forces. This would be exact in the case of a particle initially moving at the Brownian velocity and slowing to a stop. The Brownian velocity represents the relative velocity between a nanoparticle and base fluid within a nanofluid and is given by the following:

(56) $$u_{B}=\frac{1}{d_{p}}\sqrt{\frac{18k_{b}T}{\pi \rho_{p}d_{p}}}.$$

The distance between nanoparticles ξ can be estimated by considering a cubical volume of the nanofluid containing only a single nanoparticle and the surrounding base fluid. Here it is assumed that the nanoparticles are distributed homogeneously and do not interact with one another. The expression for the distance between nanoparticles is given as follows:

(57) $$\xi =\sqrt[{3}]{\frac{\pi }{6\phi }}d_{p}.$$

The Masoumi et al. [50] model for effective viscosity operates under several assumptions regarding the free stream condition and shear stress over the nanoparticles. The correction factor C was introduced to account for those simplifications and assumptions. When the free stream conditions of a fluid are far away from the particle in consideration, the properties of the viscous flow are difficult to determine. So, the assumption of creeping flow for the base fluid over the nanoparticle was made. However, this facilitates the need for a correction that considers the actual shear stress experienced by nanoparticles. The correction factor accounting for the assumption regarding the distance between the free steam and the nanoparticle is calculated as:

(58) $$C=\frac{\left[\left(-1.133\times 10^{-6}\right)d_{p}-2.771\times 10^{-6}\right]\phi +\left(9\times 10^{-8}\right)d_{p}-3.93\times 10^{-7}}{\mu_{bf}}.$$

The coefficients were determined from a limited amount of previously collected experimental data on 13 nm and 28 nm Al_2_O_3_–water nanofluid. The model, in general, has the following restriction on concentration:

(59) $$\phi <\frac{\left(-9\times 10^{-8}\right)d_{p}+3.93\times 10^{-7}}{\left(-1.133\times 10^{-6}\right)d_{p}-2.771\times 10^{-6}}.$$

Since ϕ≥0, a minimum restriction on $d_{p}$ can be derived:

(60) $$d_{p}>4.37\;\mathrm{nm}.$$

Hosseini et al. [51] created an empirical model that expresses nanofluid viscosity as a function of dimensionless groups that include the effects of temperature, particle size and volume fraction, viscosity of the base liquid, and nanolayer properties. The Hosseini et al. [51] model concludes that the viscosity of a nanofluid can be expressed as the following expression:

(61) $$\mu_{nf}=\mu_{bf}\left(\exp\left[0.72-0.485\left(\frac{T}{T_{0}}\right)+14.94\phi {\left(\frac{d_{p}+2\delta_{nl}}{d_{p}}\right)}^{3}+0.0105\left(\frac{d_{p}}{1+\delta_{nl}}\right)\right]\right),$$

where $T_{0}=20\;{}^{\circ}C$ is the reference temperature. The constants were determined based on the properties of the system, including the nanoparticles, base fluids, and their interactions. In addition, previously collected experimental data of alumina–water nanofluids and least-squares regression was used. Due to the large, negative coefficient of the temperature dimensionless group, this model predicts a nanofluid viscosity less than that of the base fluid at high temperatures. This fact will be evident shortly.

Klazly and Bognár [52] proposed a model that would consider the effects of characteristics such as the nanoparticle diameter, base fluid, type of nanoparticle, temperature, and density. Starting from existing theoretical viscosity correlations and then experimentally determined viscosity correlations for different types of nanoparticles, this model takes into consideration 1200 experimental and 4000 theoretical values collected for 50 types of nanofluids suspended in several different base fluids, with nanoparticles ranging in diameters from 2 to 300 nm and volume concentrations up to 10.0% between temperature limits of 273 and 333 K. The developed expression for calculating the effective viscosity of a nanofluid while considering both the static and dynamic states was found and is given as the following:

(62) $$\mu_{nf}=\mu_{bf}\left(\frac{\sqrt{\frac{18k_{b}T\rho_{p}}{\pi }}}{39.6231\sqrt[{3}]{\frac{\pi }{\phi }}C\sqrt{d_{p}}}+1+9.4974\phi_{e}+77.811\phi_{e}^{2}+0.9514\phi_{e}^{3}\right),$$

where $\phi_{e}$ is the effective nanoparticle volume fraction, and C is a correction factor that depends on the nanofluid volume fraction and temperature. The constants were determined from 21 viscosity correlations considering 4000 values using a regression analysis. It was assumed the intrinsic viscosity can be represented by a second-order approximation using the effective volume fraction. The effective nanoparticle volume fraction is calculated as a function of the nanoparticle volume concentration, thickness of the nanolayer, and radius of the nanoparticle, and is represented by the following equation:

(63) $$\phi_{e}=\phi \beta_{nl}^{3},$$

where $\beta_{nl}$ is the ratio of the thickness of the nanolayer to the radius of the nanoparticle plus the value one. Upon evaluating the experimental results, a correction factor C was established to take into consideration the nanofluid volume fraction, temperature, and the nanoparticle material. The correction factor can be calculated as:

(64) $$C=\left(2\times 10^{-16}\right)T^{-B_{1}}+\left(3\times 10^{-8}\right)\phi^{-B_{2}},$$

where $B_{1}$ and $B_{2}$ are constants determined by experimental data and will change based on the type of nanoparticle in use. There are three defined cases for $B_{1}$ and $B_{2}$. First, $B_{1}=3.95$ and $B_{2}=0.64$ for 40 nm particles with Al_2_O_3_, CuO, Fe, Fe_3_O_4_, SiC, ZnO, AIN, TiO_2_, SWCNT, Ag, SiO_2_, MWCNT, or DWCNT as the material. For 20 nm SWCNT, CaCO_3_, hBN, or MgO particles, $B_{1}=2$ and $B_{2}=0.64$. Otherwise, for CNT, graphite, or Cu particles of no specified size, $B_{1}=3.95$ and $B_{2}=69$.

**Figure 17:**
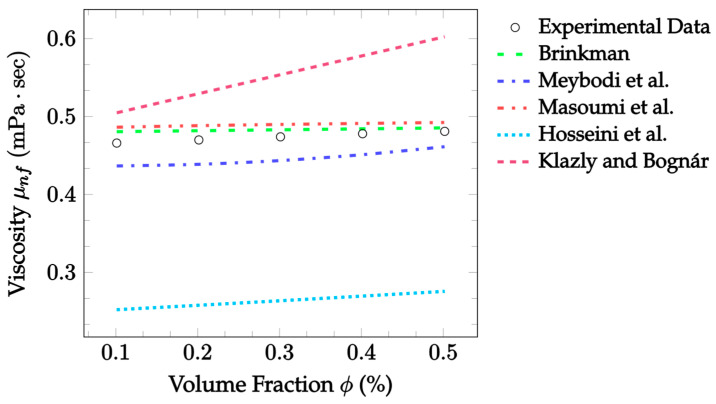
Experimental findings for Fly ash–water nanofluid compared with theoretical predictions: Experimental Data [107], Brinkman [48], Meybodi et al. [49], Masoumi et al. [50], Hosseini et al. [51], Klazly and Bognár [52].

Figure 17 presents the five previously described viscosity models compared to experimental data collected by Kanti et al. [107] for a fly ash–water nanofluid at varying volume fractions. Of the models considered, the Brinkman [48] and Masoumi et al. [50] models performed best in predicting the viscosity of the nanofluid, both overestimating slightly with MAE’s of 1.960% and 3.351%, respectively. The Hosseini et al. [51] model considerably underestimated the viscosities observed, resulting in an MAE of 44.43%. Moreover, Meybodi et al. [49] also underestimated the nanofluid viscosity and had an MAE of 13.34%. Meanwhile, the Klazly and Bognár [52] model overestimated the nanofluid viscosity. Additionally, it inaccurately predicted the rate at which the nanofluid viscosity would increase with the volume concentration. This resulted in an MAE of 16.795%.

The level of accuracy of each of the presented models compared to seven different experiments is shown below in Table 8. The Meybodi et al. [49] model had the greatest success modeling the viscosity, while the Hosseini et al. [51] model performed less successfully. The Hosseini et al. [51] model could have faced challenges due to the limited experimental analysis focusing on only one nanofluid performed in the development of the viscosity model. In general, all five of the models had difficulty predicting the viscosity for nanofluids with larger volume concentrations and diameters. However, they seemed to perform better at higher temperatures. The effect of the base fluid material did not appear to heavily influence the accuracy of the five models. The Klazly and Bognár [52] model performed significantly better when predicting the behavior of small diameter nanofluids and struggled to capture the measured results with large nanoparticle diameters. The Masoumi et al. [50] model predicted the data with a level of accuracy second to Meybodi et al. [49] across the seven experiments. The varied factors of nanoparticle diameter, volume fraction, or temperature appeared to have little correlation with accuracy of the model, making it consistent overall. The Brinkman [48] model, although only considering nanoparticle volume fraction as an input, modeled the experimental data more accurately than two others in general and performed better for smaller volume fractions, apart from the SWCNT–water nanofluid. The Brinkman [48] and Masoumi et al. [50] models tend to make predictions nearly identical to one another for each of the seven experiments considered.

## 5. Optimization of Nanofluids for Heat Transfer Applications

**Figure 18:**
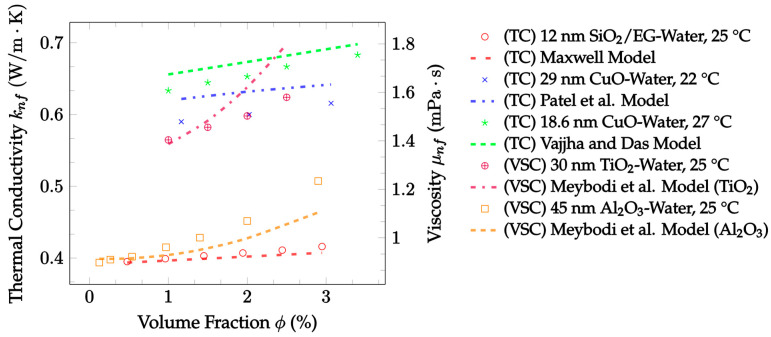
Thermal conductivity and viscosity of various nanofluids as a function of volume fraction with corresponding theoretical predictions: (TC) 12 nm SiO_2_/EG–water, 25 °C [109], (TC) Maxwell Model [14], (TC) 29 nm CuO–water, 22 °C [66], (TC) Patel et al. Model [23], (TC) 18.6 nm CuO–water, 27 °C [12], (TC) Vajjha and Das Model [22], (VSC) 30 nm TiO_2_–water, 25 °C, [111], (VSC) Meybodi et al. Model (TiO_2_) [49], (VSC) 45 nm Al_2_O_3_–water, 25 °C [112], (VSC) Meybodi et al. Model (Al_2_O_3_) [49].

In most cases, it was observed that adding nanoparticles into the base liquid or raising the nanoparticle concentration would increase the thermal conductivity and viscosity of the nanofluid simultaneously. These effects are visible in Figure 18 theoretically and experimentally. Both theoretical models and experimental data show that the thermal conductivity and viscosity of nanofluids increase with nanoparticle concentration in most cases. Thermal conductivity enhancement counts as a positive impact, while viscosity enhancement counts as a negative side effect on the heat transfer coefficient. As viscosity increases, the random motion of nanoparticles and molecules decreases. Consequently, mass transfer and energy transfer from one layer to another would decrease, which has a negative impact on the heat transfer coefficient. Therefore, it is necessary to optimize the effects of nanoparticles on the thermal physical properties of base liquids to maximize the heat transfer coefficient. In this section, the optimization of nanofluids for use in energy applications is briefly reviewed. This paper takes advantage of both theoretical and experimental data from independent sources. Through this data, the combined effects of temperature and concentration can be analyzed. From here, a recommendation can be made on how to engineer a nanofluid to achieve effective thermal management.

When designing a nanofluid, it is important to account for all the necessary properties to maximize efficiency. For instance, one nanofluid may have a significantly high thermal conductivity. This would provide a net increase in the heat transfer coefficient for a given fluid channel. However, the same fluid may also inherit a significantly high viscosity. In this case, the greater fluid flow resistance would require more power for the pump, and the random motion of nanoparticles would decrease. Consequently, the benefits of the thermal conductivity enhancement are completely negated due to the excess input work required. So, in order to optimize the nanofluid, the thermal conductivity must be maximized, and the viscosity minimized for operating conditions.

Figure 18 shows various sets of data for the thermal conductivity and viscosity of nanofluids as a function of concentration theoretically and experimentally. Theoretical models are compared with experimental data to show the effects of nanoparticle concentration on the thermal conductivity and viscosity of different nanofluids. The thermal conductivity of 12 nm SiO_2_ nanoparticles suspended in a 60:40 EG–water mixture was measured by Dong et al. [109]. It was found that at a concentration of approximately 3%, the thermal conductivity was enhanced by up to 6.4% compared to the base liquid. The Maxwell [14] model’s predictions are in good agreement with this finding, yielding a high level of accuracy (1.176%). Similar experiments were performed on 29 nm CuO–water nanofluid by Mintsa et al. [66]. Their observations aligned with Dong et al. [109], as it was found that the thermal conductivity was enhanced by approximately 5.0% at a concentration of around 3%. The Patel et al. [23] model was used to predict this dataset, and it had a relatively low error of 4.997%. Furthermore, Lee et al. [12] measured the thermal conductivity of 18.6 nm CuO–water. They found that there was an enhancement of 12.02% at a concentration of 3.4%. In this case, the Vajjha and Das [22] model agreed, accurate within 2.847% on average. Together, these theoretical predictions and experimental findings show that the addition of nanoparticles enhances the thermal conductivity of the fluid. This is a net benefit for heat transfer.

Viscosity seems to show a similar effect with respect to concentration. Hussein et al. [111] studied the viscosity of their 30 nm TiO_2_–water nanofluid. They found that at a concentration of 2.5%, the viscosity increased by 21.5%. Similarly, Pastoriza-Gallego et al. [112] experimented with 45 nm Al_2_O_3_ nanofluid and observed the same effect. At a concentration of 2.9%, the viscosity had increased by nearly 38.5%. In both cases, the predictions of Meybodi et al. [49] agree with the recognized trend. The model had an error of 5.635% and 5.126% for the TiO_2_ and Al_2_O_3_ nanofluids, respectively. Again, this net increase in viscosity is a downside of adding nanoparticles. It results in the requirement of more power for the pump, and thus negates the benefits of increased thermal conductivity. However, it is reminded that the goal in mind is to design an optimal nanofluid. Therefore, it is necessary to outline the effects of more parameters (temperature) to gain better insight.

**Figure 19:**
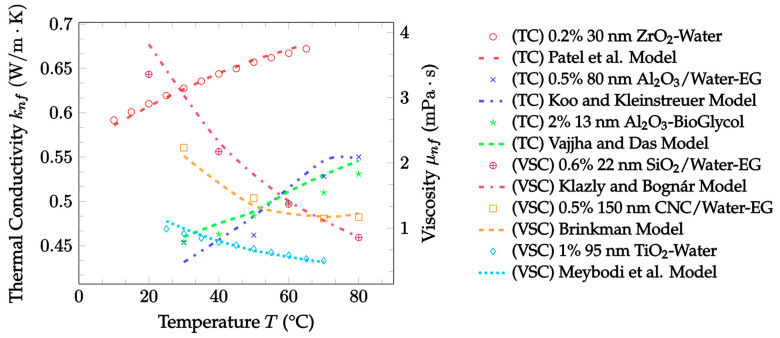
Thermal conductivity and viscosity of nanofluids as a function of temperature with additional theoretical correlations: (TC) 0.2% 30 nm ZrO_2_–water [65], Patel et al. Model [23], (TC) 0.5% 80 nm Al_2_O_3_/Water–EG [113], (TC) Koo and Kleinstreuer Model [21], (TC) 2% 13 nm Al_2_O_3_-BioGlycol [114], (TC) Vajjha and Das Model [22], (VSC) 0.6% 22 nm SiO_2_/EG–water [115], (VSC) Klazly and Bognár Model [52], (VSC) 0.5% 150 nm CNC/Water–EG [113], (VSC) Brinkman Model [48], (VSC) 1% 95 nm TiO_2_–water [116], (VSC) Meybodi et al. Model [49].

Figure 19 outlines the effect of temperature on the thermal conductivity and viscosity of different nanofluids. This figure shows that as temperature increases, thermal conductivity increases and viscosity decreases for most nanofluids with different concentrations. In fact, temperature magnifies the positive side effects of nanoparticles on the thermal conductivity, and decreases or eliminates the negative side effects on viscosity. In the case of nanofluid flows in microchannels, we need to design the operating temperature in a way that maximizes the positive effects of nanoparticles on thermal conductivity and minimizes their negative effects on viscosity. These optimizations would work together to maximize the heat transfer coefficient. It is necessary to optimize the operating temperature and characteristics of nanofluids to maximize the heat transfer coefficient in channels. Çolak [65] measured the thermal conductivity of 0.2% 30 nm ZrO_2_–water nanofluid. It was found that the nanofluid thermal conductivity increases with an increasing temperature. Specifically, an enhancement of 13.6% occurred when going from 5 °C to 60 °C. The predictions of Patel et al. [23] are in excellent agreement with this data, showing a high accuracy of 0.461%. This effect was repeated in the experiments carried out by Farhana et al. [113], who examined 80 nm Al_2_O_3_ nanoparticles in a 60:40 water–EG base fluid. They noted a 21.2% improvement in the thermal conductivity for the 80 °C fluid compared to the 30 °C fluid. These findings are solidified by the Koo and Kleinstreuer [21] model, which gave an accuracy of 3.351%. Moreover, Abdolbaqi et al. [114] investigated the thermal conductivity of 13 nm Al_2_O_3_–BioGlycol nanofluid. Again, a similar conclusion was reached. It was found that over a temperature range of 30 °C to 80 °C, the thermal conductivity observed a 17.0% enhancement. For this nanofluid, the Vajjha and Das [22] model was found to agree within an error of 2.37%. From this analysis, it can be concluded that temperature has the same effect on the thermal conductivity as concentration. Namely, the thermal conductivity increases as temperature increases.

Now, the effect of temperature on nanofluid viscosity is investigated. Lahari et al. [115] investigated the viscosity of 22 nm SiO_2_/70:30 water–EG nanofluid with a 0.6% concentration. Unlike what was previously described with the effects of concentration, they found that viscosity decreased with increasing temperatures. Specifically, viscosity decreased by 74.47% in the 80 °C fluid compared with the original 20 °C fluid. Here the Klazly and Bognár [52] model was in good agreement with these results, yielding an accuracy of 6.216%. Farhana et al. [113] also measured the viscosity of their nanofluid, which used carbon nanocrystals (CNC) suspended in a 60:40 water–EG mixture. Likewise, they found that nanofluid viscosity decreased by 47.7% when going from 30 °C to 80 °C. The predictions made by Brinkman [48] agree here, giving a level of accuracy of 5.176%. Lastly, Bahiraei et al. [116] investigated the viscosity of a 95 nm TiO_2_–water nanofluid with a 1% concentration. Their findings were no different, showing that viscosity decreased by 49.4% when increasing the temperature from 25 °C, to 70 °C. The Meybodi et al. [49] model goes along with this finding, having an MAE of 4.881%. All of these data agree that temperature has the opposite effect on viscosity as concentration. It is also noted that at high temperatures, the effect of concentration on viscosity seems to be greatly reduced. Specifically, the nanofluids with different particle concentrations at low temperatures spread over the range of 1 to 4 mPa · s. At higher temperatures, this spread is reduced to the region from approximately 0.5 to 1.2 mPa · s. Therefore, adding more nanoparticles could have less of a drawback at higher temperatures.

From the results of this brief review, there is a clear method to optimize nanofluids for applications in energy transport. The idea would be to increase the temperature of the nanofluid to the high temperature range. Firstly, increasing the temperature simultaneously enhances the nanofluid’s thermal conductivity and decreases its viscosity. This is excellent for heat transfer systems. The thermal conductivity enhancement allows for more efficient heat transfer through the fluid, while the decrease in viscosity lowers the amount of pumping power necessary. Secondly, it may be feasible to add more nanoparticles to the fluid, as it was observed they may have less of an effect on the viscosity at these temperatures. This is desirable, since it was also found that increasing the concentration increased the thermal conductivity of the nanofluid. Fortunately, this solution is feasible in many different applications. Electronics, for instance, frequently operate in the 60 °C to 80 °C range. This makes nanofluids prime candidates to act as working fluids in these systems.

## 6. Conclusions

The purpose of this paper is to develop a method to predict the thermal conductivity of hybrid nanofluids. This paper reviewed the current theoretical models to predict the thermal conductivity and viscosity of nanofluids. The various enhancement mechanisms, advantages and disadvantages of each model, and the level of accuracy of each model were all discussed in detail. In spite of demand, it was found that little work has been done on the development of thermal conductivity models for general hybrid nanofluids. Therefore, in this research, a new model was developed to predict the thermal conductivity of hybrid nanofluids, ternary hybrid nanofluids, and so on. Additionally, the optimization of nanofluids for microfluidic applications in thermal management was explored.

The models based in Effective Medium Theory are some of the earliest and most fundamental. It was observed that Jeffery’s model [16] had the highest level of accuracy for given, independent experimental data. The minimum, maximum, and average errors were observed to be 0.547%, 22.661%, and 6.383%, respectively, for given independent data. Brownian models tend to use parameters such as the base fluid density, viscosity, specific heat, and nanoparticle size. It was found that the lowest error of all the Brownian models was 0.0624% for given independent experimental data, which was predicted by Patel et al. [23]. In addition, the maximum and average errors were determined to be 14.88% and 3.948%, respectively, in this category. Given that these values are less than those observed for EMT models, it can be concluded that Brownian motion models can predict the thermal conductivity enhancement slightly better. It is obvious that the random motion of nanoparticles has an important role in transferring energy from one layer to another. The interfacial layer is another mechanism commonly cited as a reason behind thermal conductivity enhancement. It was observed that the minimum error was 0.369%, predicted by Yu and Choi [27] for given independent experimental data. Simultaneously, the maximum predicted error was 41.689%, and the average error was 7.368%. Aggregation models have a major advantage, as they assume that the nanoparticles interact with one another and aggregate in the suspension. It was determined that the minimum error was predicted by Prasher et al. [29] at 2.204%. In addition, the maximum and average errors were determined to be 87.23% and 18.94%, respectively, in this category. Molecular dynamics simulations have also been applied to predict the nanofluid thermal conductivity. The basic idea is to simulate all the interactions between every particle in a nanofluid system. The major benefit of this technique is that the resulting models can take into account many different mechanisms comprehensively. Again, assumptions were still required to be able to compare these models to given data. It was found that the error reached its minimum of 1.409% in a prediction by Mirmohammadi et al. [36]. The highest and average errors for the molecular dynamics models were found to be 49.539% and 7.759%, respectively.

The focus of this paper was on the development of models for hybrid nanofluids. A large portion of the existing correlations are for a hybrid nanofluid with a given particle material, base fluid, and particle mixing ratio. Due to the narrow validity ranges of these models, their level of accuracy with respect to independent data could not be viably measured and compared. Thus, the remaining portion of general hybrid nanofluid models were chosen for analysis. It was found that these models were typically based on existing mono nanofluid models. In addition, the considered mechanisms were Brownian motion and EMT. It was determined that the Devi and Devi [38] model yielded the minimum error compared against given independent experimental data, giving a value of 1.763%. Furthermore, the maximum error was determined to be 20.069%, and the average error was 8.403%.

Given that a majority of hybrid nanofluid correlations are empirical and have narrow validity ranges, it seems that the theory of hybrid nanofluid thermal conductivity has fallen behind experimental work. Thus, we propose a new general model to predict the thermal conductivity of hybrid nanofluids. In addition, it is applicable to nanofluids with any number of distinct nanoparticles. For independent data of a hybrid nanofluid, the most accurate prediction was found to have an error of 0.393%. Meanwhile, the previous most accurate model yielded an error of 1.763%. This pattern was consistent for nearly all independent data analyzed. Moreover, this model also accurately predicted independent experimental data for ternary hybrid nanofluids. It was found that the minimum error for these fluids was 0.831%. The average error of the new model when compared with given experimental data for hybrid nanofluids was found to be 5.289%. This was lower than the average error of the best preexisting model.

Finally, the optimization of characteristics of nanofluids for microfluidic applications was examined. It was first found that introducing nanoparticles into the base liquid simultaneously increases the thermal conductivity and the viscosity of the working fluid. The thermal conductivity effect is beneficial, as the more efficient energy transfer through the fluid would allow for an increased heat transfer coefficient in fluid channels. The viscosity enhancement, however, is a negative side effect. The random motion of the particles and molecules inside the nanofluid decreases, and as a result, the rate of energy transfer decreases. Therefore, it is required that these effects are optimized in order to properly take advantage of the effects of nanoparticles. It was observed that as the operating temperature increases, the negative side effect of nanoparticles diminishes, while the nanofluid thermal conductivity increases. Thus, the operating temperature and characteristics of the nanofluid should be optimized to maximize the nanofluid heat transfer coefficient in microchannels. This phenomenon was investigated by comparing theoretical predictions with given experimental data, for both thermal conductivity and viscosity.

## Figures and Tables

**Table 1:** Level of accuracy (%) of each EMT model when compared against the given experimental data.

Dataset	Maxwell [14]	Hamilton and Crosser [15]	Timofeeva et al. [17]	Jeffery [16]	Sundar et al. [18]
${\mathrm{Al}}_{2}O_{3}–\mathrm{water},\;300\;K,\;1–4.3\%,\;n=3$ [12] *	1.626	1.626	1.719	2.216	3.698
CuO–water, 300 K, 1–3.4%, n=3 [12]	1.684	1.684	1.253	0.844	5.086
${\mathrm{Al}}_{2}O_{3}–\mathrm{EG},\;300\;K,\;1–5\%,\;n=3\;$[12]	0.933	0.933	1.072	0.681	6.524
CuO–EG, 300 K, 1–5%, n=3, [12]	4.205	4.205	4.114	3.750	8.522
${\mathrm{Al}}_{2}O_{3}–\mathrm{water},\;300\;K,\;1–4\%,\;n=3$, [59]	1.501	1.501	1.612	2.012	3.127
CuO–water, 300 K, 1–3.5%, n=3 [59] **	1.379	1.379	0.941	0.547	4.824
CuO–EG, 300 K, 1–5%, n=3 [60]	4.229	4.229	4.172	3.671	9.436
${\mathrm{Al}}_{2}O_{3}–\mathrm{water},\;300\;K,\;2–10\%,\;n=3$ [61]	8.984	8.984	8.343	10.193	4.433
CuO–water, 300 K, 4–6%, n=3 [61]	15.482	15.482	14.917	13.673	22.661
CNT–water, 300 K, 0.1–0.9%, n=9.35 [62]	15.854	13.505	15.860	15.850	16.571

* Experimental data used in Figure 1; ** Experimental data used in Figure 2.

**Table 2:** Level of accuracy (%) of each Brownian model when compared against given experimental data.

Dataset	Corcione [19]	Chon et al. [20]	Koo and Kleinstreuer [21]	Vajjha and Das [22]	Patel et al. [23]	Kumar et al. [24]
Al_2_O_3_–water, 300 K, 24.4 nm, 1–4.3% [12] ^†^	4.051	4.831	8.456	5.519	5.243	6.318
CuO–water, 300 K, 18.6 nm, 1–3.4% [12]	2.180	6.223	14.88	2.847	1.804	6.994
Al_2_O_3_–EG, 300 K, 24.4 nm, 1–5% [12] *	6.675	9.063	0.884	0.867	5.456	9.067
CuO–EG, 300 K, 18.6 nm, 1–4%, [12] ^†^	8.370	10.65	3.545	4.021	2.719	10.651
Al_2_O_3_–water, 22 °C, 36 nm, 1–3% [66]	3.178	3.137	5.614	4.237	4.278	3.990
Al_2_O_3_–water, 22 °C, 47 nm, 1–3% [66]	1.720	3.171	5.202	3.959	3.882	3.952
CuO–water, 22 °C, 29 nm, 1–3% [66] ^†^	4.316	1.984	11.96	6.063	4.997	2.572
ZrO_2_–water, 10 °C, 30 nm, 0.0125–0.2% [65]	0.653	1.078	1.172	0.541	0.496	1.085
ZrO_2_–water, 35 °C, 30 nm, 0.0125–0.2% [65] **	0.114	1.072	0.676	4.017	0.0624	1.088
ZrO_2_–water, 65 °C, 30 nm, 0.0125–0.2% [65]	1.832	1.052	2.994	8.578	0.493	1.088
ZrO_2_–water, 10–65 °C, 30 nm, 0.2% [65] ***	1.371	1.922	3.441	3.807	0.461	1.964

* Experimental data used in Figure 3; ** Experimental data used in Figure 4; *** Experimental data used in Figure 5; ^†^ Experimental data used in Figure 6.

**Table 3:** Level of accuracy (%) of each nanolayer model when compared against given experimental data.

Dataset	Leong et al. [25]	Xie et al. [26]	Yu and Choi [27]	Tinga et al. [28]
TiO_2_–water, 15 °C, 21 nm [70]	1.154	3.351	2.681	4.144
TiO_2_–water, 25 °C, 21 nm [70]	4.156	6.179	5.558	6.910
TiO_2_–water, 35 °C, 21 nm, [70]	1.644	1.658	1.298	3.538
CuO–water, 22 °C, 29 nm [66]	26.65	9.634	13.42	2.328
Al_2_O_3_–water, 22 °C, 36 nm, [66]	35.18	12.20	16.22	2.320
Al_2_O_3_–water, 22 °C, 47 nm [66]	41.689	13.721	17.681	5.240
Al_2_O_3_–water, 27 °C, 24.4 nm [12]	8.654	0.650	2.476	2.727
CuO–water, 27 °C, 18.6 nm [12]	2.874	2.478	0.817	3.965
Al_2_O_3_–EG, 27 °C, 24.4 nm [12] *	6.808	1.900	0.369	5.382
CuO–EG, 27 °C, 18.6 nm [12]	1.769	5.158	3.121	7.034

* Experimental data used in Figure 7.

**Table 4:** Level of accuracy (%) of each aggregate model when compared against given experimental data.

Dataset	Prasher et al. [29]	Xuan et al. [30]	Feng et al. [31]	Wang et al. [32]
CuO–EG, 25 °C, 10 nm, 0.18%, 14–48 m, [73] *	2.204	2.578	3.643	3.409
Fe_3_O_4_–water, 25 °C, 9.8 nm, 1–3%, 5–50 nm [74] **	9.442	4.377	16.27	14.45
Fe_3_O_4_-Toulene, 24 °C, 12.4 nm, 0.01–0.15%, 15–39 nm [75]	4.573	2.675	5.063	4.950
Al_2_O_3_–water, 25 °C, 30 nm, 5%, 68–74 nm [76]	87.23	39.31	9.829	18.41
Al_2_O_3_–water, 50 °C, 30 nm, 5%, 68–74 nm [76]	82.02	44.53	7.686	16.13

* Experimental data used in Figure 8; ** Experimental data used in Figure 9.

**Table 5:** Level of accuracy (%) of each MDS model when compared against given experimental data.

Dataset	Evans et al. [33]	Vladkov and Barrat [34]	Lin et al. [35]	Mirmohammadi et al. [36]
$\mathrm{CuO}–\mathrm{Ethanol},\;25\;{}^{\circ}C,\;6.18\times 10^{-4}-0.637\%$ [78] *	2.947	2.891	2.827	1.409
$\mathrm{CuO}–\mathrm{Ethanol},\;40\;{}^{\circ}C,\;5.82\times 10^{-4}$−0.626% [78]	10.518	10.471	10.041	6.459
$\mathrm{CuO}–\mathrm{Ethanol},\;55\;{}^{\circ}C,\;5.97\times 10^{-4}$−0.625% [78]	15.629	15.589	15.540	11.140
${\mathrm{TiO}}_{2}–\mathrm{Ethanol},\;25\;{}^{\circ}C,\;9.24\times 10^{-4}-$0.957% [78] **	2.979	2.863	2.724	3.249
${\mathrm{TiO}}_{2}–\mathrm{Ethanol},\;40\;{}^{\circ}C,\;9.24\times 10^{-4}-$0.957% [78]	9.456	9.356	9.235	3.458
Al_2_O_3_–water, 30 °C, 20 nm, 0.125–0.511% [79]	7.464	7.223	7.009	4.844
Al_2_O_3_–water, 30 °C, 50 nm, 0.125–0.511% [79]	3.327	3.203	3.104	6.761
Al_2_O_3_–water, 30 °C, 100 nm, 0.125–0.511% [79] ***	2.013	1.957	1.906	6.490
Al_2_O_3_–water, 23 °C, 40 nm, 2.5–10.0% [17]	2.084	1.946	3.325	49.539
Al_2_O_3_–EG, 23 °C, 40 nm, 1.0–10.0% [17]	1.913	1.484	2.568	43.430

* Experimental data used in Figure 10; ** Experimental data used in Figure 11; *** Experimental data used in Figure 12.

**Table 6:** Level of accuracy (%) of each hybrid nanofluid model when compared against given experimental data.

Dataset	Chamkha et al. [37]	Devi and Devi [38]	Chougule and Sahu [39]
50:50 69 nm TiO_2_-40 nm CaCO_3_/Water, 30 °C, 0.05–0.25% [88] *	1.972	1.763	3.730
80:20 29 nm Fly Ash-50 nm Cu/Water, 30 °C, 0.5–4% [80]	19.793	18.948	20.069
80:20 37.5 nm Y_2_O_3_-50 nm MWCNT/Water, 30 °C, 0.01–0.2% [89]	1.929	2.028	1.896
10:1 15 nm TiO_2_-15 nm Ag/Water, 30 °C, 0.5–5% [87] **	3.377	1.952	2.074
80:20 40 nm CuO-40 nm MgO/Water, 30 °C, 0.25–1.25% [90]	4.642	3.304	5.096
60:40 20 nm MgO-20 nm ZnO/Water, 0.1%, 20–50 °C [91]	16.693	16.532	16.681
60:40 24.24 nm Fe_3_O_4_-1.1 nm SWCNT/EG, 50 °C, 0.01–0.5% [92]	14.538	11.000	14.765
50:50 20 nm Al_2_O_3_-50 nm CeO_2_, 50 °C, 0.01–5% [93]	4.934	4.650	5.004
40:25 50 nm TiO_2_-30 nm SiO_2_/60:40 Water–EG, 50 °C, 0.5–3% [94]	10.104	8.302	11.111

* Experimental data used in Figure 13; ** Experimental data used in Figure 14.

**Table 7:** Level of accuracy (%) of the best performing preexisting hybrid nanofluid model versus the present model.

Dataset	Devi and Devi [38]	Present Model
50:50 69 nm TiO_2_-40 nm CaCO_3_/Water, 30 °C, 0.05–0.25% [88] *	1.763	0.393
80:20 29 nm Fly Ash-50 nm Cu/Water, 30 °C, 0.5–4% [80]	18.95	11.31
80:20 37.5 nm Y_2_O_3_-50 nm MWCNT/Water, 30 °C, 0.01–0.2% [89]	2.028	0.397
10:1 15 nm TiO_2_-15 nm Ag/Water, 30 °C, 0.5–5% [87]	1.952	7.522
80:20 40 nm CuO-40 nm MgO/Water, 30 °C, 0.25–1.25% [90]	3.304	2.263
60:40 20 nm MgO-20 nm ZnO/Water, 0.1%, 20–50 °C [91]	16.53	12.65
60:40 24.24 nm Fe_3_O_4_-1.1 nm SWCNT/EG, 50 °C, 0.01–0.5% [92]	11.00	10.15
50:50 20 nm Al_2_O_3_-50 nm CeO_2_, 50 °C, 0.01–5% [93]	4.650	1.044
40:25 50 nm TiO_2_-30 nm SiO_2_/60:40 Water–EG, 50 °C, 0.5–3% [94]	8.302	1.868

* Experimental data used in Figure 15.

**Table 8:** Level of accuracy (%) of each nanofluid viscosity model when compared against given experimental data.

Dataset	Brinkman [48]	Meybodi et al. [49]	Masoumi et al. [50]	Hosseini et al. [51]	Klazly and Bognár [52]
TiO_2_–water, 21 nm, 40 °C, 1–5% [108]	47.91	21.78	21.95	24.48	30.48
Fly ash–water, 14 nm, 60 °C, 0.1–0.5% [107] *	1.960	13.34	5.820	44.34	16.80
Graphene-PG, 5 nm, 50 °C, 0.02–0.1% [10]	9.883	27.95	22.06	41.02	9.130
SiO_2_–EG/Water, 15 nm, 50 °C, 0.5–3% [109]	25.59	17.88	20.44	32.27	30.08
SWCNT–water, 9.2 nm, 20 °C, 0.006–0.56% [11]	17.83	21.34	14.89	33.81	13.23
Al_2_O_3_–water, 30 nm, 25 °C, 1.3–5.9% [110]	30.40	22.65	24.84	59.80	67.34
TiO_2_–water, 30 nm, 25 °C, 1–2.5% [111]	8.414	5.635	6.207	57.48	59.11

* Experimental data used in Figure 17.

## Data Availability

The data presented in this study are available on request from the corresponding author.

## Appendix A

**Table A1:** Nomenclature with standard units. Note that many models use other units for calculations.

Symbol	Definition
X	Number of data points
$k_{\mathrm{th}}$	Predicted value
$k_{\exp}$	Measured value
k	Thermal conductivity (W/m·K)
ϕ	Volume concentration of nanoparticles
n	Shape factor
ψ	Sphericity
Re	Reynolds number
Pr	Prandtl number
c	Specific heat (J/kg·K)
μ	Dynamic viscosity (kg/m·s)
ρ	Density (kg/m^3^)
T	Temperature (°C)
$T_{fr}$	Freezing temperature (K)
$T_{0}$	Reference temperature (°C)
$u_{B}$	Brownian velocity (m/s)
$k_{b}$	Boltzmann’s constant (1.380649 × 10^−23^ m^2^ kg s^−2^ K^−1^)
d	Diameter (m)
r	Radius (m)
$l_{bf}$	Mean free path of base fluid molecule (m)
β	Empirical correlation factor from Koo and Kleinstreuer [30] or Vajjha and Das [31]
$C_{u}$	Constant from Kumar et al. [33], taken as 2.95 in this paper
$\beta_{nl}$	Ratio of equivalent concentration including the nanolayer the to actual concentration
$\delta_{nl}$	Nanolayer thickness (m)
γ	Ratio of nanolayer to particle thermal conductivity
$d_{f}$	Fractal dimension
$n\left(r_{a}\right)$	Aggregate distribution function
h	Equivalent matrix thickness (m)
ϵ	Ratio of particle radius to equivalent matrix thickness
Ω	Ratio of equivalent thermal thickness to particle radius
ζ	Dimensionless thermal conductivity parameter
$A_{s}$	Surface area (m^2^)
V	Volume (m^3^)
λ	General enhancement term
N	Number of distinct nanoparticles in suspension
ξ	Average distance between nanoparticles (m)
C	Empirical correlation factor from Hosseini et al. [13] or Klazly and Bognár [14]
$B_{1},B_{2}$	Empirical correlation factors from Klazly and Bognár [14]
**Subscripts**	
nf	Nanofluid
bf	Base fluid
p	Particle
nl	Nanolayer
pe	Particle equivalent
a	Aggregate
e	Equivalent
cl	Cluster
hnf	Hybrid nanofluid
thnf	Ternary hybrid nanofluid
Nhnf	N hybrid nanofluid
1,2,…,i,…,N	Corresponding to nanoparticle in N hybrid nanofluid
**Abbreviations**	
MAE	Mean absolute error (%)
EMT	Effective Medium Theory
CNT	Carbon nanotubes
SWCNT	Single-walled carbon nanotubes
MWCNT	Multi-walled carbon nanotubes
EG	Ethylene Glycol
PG	Propylene Glycol
MDS	Molecular Dynamics Simulation(s)
TC	Thermal conductivity
VSC	Viscosity
CNC	Cellulose nanocrystals

## Appendix B

**Table A2:** Summary of nanofluid thermal conductivity models.

**Source**	**Description**	**Model**
Maxwell [14]	Modified Einstein model, assumes spherical particles.	$k_{nf}=k_{bf}\frac{k_{p}+2k_{bf}+2\phi \left(k_{p}-k_{bf}\right)}{k_{p}+2k_{bf}-\phi \left(k_{p}-k_{bf}\right)}.$
Hamilton and Crosser [15]	EMT, Modified Maxwell [14] model accounting for particle shape.	$k_{nf}=k_{bf}\frac{k_{p}+\left(n-1\right)k_{bf}-\left(n-1\right)\left(k_{bf}-k_{p}\right)\phi }{k_{p}+\left(n-1\right)k_{bf}+\left(k_{bf}-k_{p}\right)\phi },$ $n=\frac{3}{\psi }.$
Jeffery [16]	EMT, assumes static and homogeneous particles, and is a truncated infinite series.	$k_{nf}=k_{bf}\left(1+3\frac{k_{p}-k_{bf}}{k_{p}-2k_{bf}}\phi +\phi^{2}\left(3{\left(\frac{k_{p}-k_{bf}}{k_{p}-2k_{bf}}\right)}^{2}+\frac{3}{4}{\left(\frac{k_{p}-k_{bf}}{k_{p}-2k_{bf}}\right)}^{3}+\frac{9}{16}{\left(\frac{k_{p}-k_{bf}}{k_{p}-2k_{bf}}\right)}^{3}\left(\frac{\frac{k_{p}}{k_{bf}}+2}{2\frac{k_{p}}{k_{bf}}+3}\right)\right)\right).$
Timofeeva et al. [17]	EMT, as-sumes uses *k**_p_* ≫ *k**_bf_*, Al_2_O_3_–water data.	$k_{nf}=k_{bf}\left(1+3\phi \right).$
Sundar et al. [18]	EMT, modified Timofeeva et al. [17] model using Fe_3_O_4_–water data.	$k_{nf}=k_{bf}{\left(1+10.5\phi \right)}^{0.1051}.$
Corcione [19]	Brownian, empircal correlation, wide range of input data, considers Brownian motion through Reynolds number.	knf=(1+4.4Re0.4Pr0.66(TTfr)10(kpkbf)0.03ϕ0.66)kbf, $\mathrm{Pr}=\frac{c_{bf}\mu_{bf}}{k_{bf}},$ Re=ρbfuBdpμbf, $u_{B}=\frac{2k_{b}T}{\pi \mu_{bf}d_{p}^{2}}.$
Chon et al. [20]	Brownian, empirical correlation using Buckingham *π* Theorem and Al_2_O_3_–water data.	knf=(1+64.7ϕ0.746(dbfdp)0.369(kpkbf)0.746Pr0.9955Re1.2321)kbf, Re=ρbfkbT3πμbf2lbf.
Koo and Kleinstreuer [21]	Brownian, accounts for static and dynamic enhance-ment, β given in Table 2.	$k_{nf}=\left(\frac{k_{p}+2k_{bf}-2\phi \left(k_{bf}-k_{p}\right)}{k_{p}+2k_{bf}+\phi \left(k_{bf}-k_{p}\right)}+5\times 10^{4}\beta \phi \rho_{bf}c_{bf}\sqrt{\frac{k_{b}T}{\rho_{p}d_{p}}}f\left(T,\phi \right)\right)k_{bf},$ f(T,ϕ)=(−6.04ϕ+0.4705)T+(1722.3ϕ−134.63).
Vajjha and Das [22]	Brownian, validity range extension of Koo and Kleinstreuer [21] model, β in Table 3.	$f\left(T,\phi \right)=\left(0.028217\phi +0.003917\right)\left(\frac{T}{T_{0}}\right)-\left(0.030669\phi +0.00391123\right).$
Patel et al. [23]	Brownian, non- linear regression analysis over wide range of data.	$k_{nf}=\left(1+0.135{\left(\frac{k_{p}}{k_{bf}}\right)}^{0.273}\phi^{0.467}{\left(\frac{T}{20}\right)}^{0.547}{\left(\frac{100}{d_{p}}\right)}^{0.234}\right)k_{bf}.$
Kumar et al. [24]	Brownian, combination of static and dynamic enhance- ment models.	$k_{nf}=\left(1+C_{u}u_{B}\frac{\phi r_{bf}}{k_{bf}\left(1-\phi \right)r_{p}}\right)k_{bf}.$
Leong et al. [25]	Nanolayer, assumes spherical particles and steady-state heat transfer, derived from cylindrical 2D heat conduction equation.	$k_{nf}=\frac{\left(k_{p}-k_{nl}\right)\phi k_{nl}\left[2{\left(1+\frac{\delta_{nl}}{2r_{p}}\right)}^{3}-{\left(1+\frac{\delta_{nl}}{r_{p}}\right)}^{3}+1\right]+\left(k_{p}+2k_{nl}\right){\left(1+\frac{\delta_{nl}}{2r_{p}}\right)}^{3}\left[\phi {\left(1+\frac{\delta_{nl}}{2r_{p}}\right)}^{3}\left(k_{nl}-k_{bf}\right)+k_{bf}\right]}{{\left(1+\frac{\delta_{nl}}{2r_{p}}\right)}^{3}\left(k_{p}+2k_{nl}\right)-\left(k_{p}-k_{nl}\right)\phi \left[{\left(1+\frac{\delta_{nl}}{2r_{p}}\right)}^{3}+{\left(1+\frac{\delta_{nl}}{r_{p}}\right)}^{3}-1\right]}.$
Xie et al. [26]	Nanolayer, assumes *k**_nl_* varies linearly along the nanolayer thickness, utilizes order of magnitude analysis.	$k_{nf}=k_{bf}\left(3\Theta \phi +\frac{3\Theta^{2}\phi^{2}}{1-\Theta \phi }+1\right),$ $\Theta =\frac{\left(\frac{k_{nl}-k_{bf}}{k_{nl}+2k_{bf}}\right)\left[{\left(1+\frac{\delta_{nl}}{r_{p}}\right)}^{3}-\left[\frac{\left(k_{p}-k_{nl}\right)\left(k_{bf}+2k_{nl}\right)}{\left(k_{p}+2k_{nl}\right)\left(k_{bf}-k_{nl}\right)}\right]\right]}{{\left(1+\frac{\delta_{nl}}{r_{p}}\right)}^{3}+2\left[\left(\frac{k_{nl}-k_{bf}}{k_{nl}+2k_{bf}}\right)\left(\frac{k_{p}-k_{nl}}{k_{p}+2k_{nl}}\right)\right]}.$
Yu and Choi [27]	Nanolayer, modified Maxwell [14] model by replacing particle properties with effective ones. Assumes spherical particles in a homogeneous distribution.	$k_{nf}=\left(\frac{k_{pe}+2k_{bf}+2\left(k_{pe}-k_{bf}\right){\left(\beta_{nl}\right)}^{3}\phi }{k_{pe}+2k_{bf}-\left(k_{pe}-k_{bf}\right){\left(\beta_{nl}\right)}^{3}\phi }\right)k_{bf},$ $k_{pe}=\left(\frac{\left[2\left(1-\gamma \right)+{\left(\beta_{nl}\right)}^{3}\left(1+2\gamma \right)\right]\gamma }{-\left(1-\gamma \right)+{\left(\beta_{nl}\right)}^{3}\left(1+2\gamma \right)}\right)k_{p},$ $\beta_{nl}=1+\frac{\delta_{nl}}{r_{p}},$ $\gamma =\frac{k_{nl}}{k_{p}}.$
Tinga et al. [28]	Nanolayer, original model for prediction of dielectric constant of cellulose-air–water mixtures, assumes spherical particles.	$k_{nf}=\left(1+\frac{3\phi \left[\left(\beta_{nl}{}^{3}-1\right)\left(2k_{nl}+k_{p}\right)\left(k_{nl}-k_{bf}\right)-\left(k_{nl}-k_{p}\right)\left(2k_{nl}+k_{bf}\right)\right]}{\left(2k_{bf}+k_{nl}\right)\left(2k_{nl}+k_{p}\right)-\left(\frac{2}{\beta_{nl}{}^{3}-1}\right)\left(k_{nl}-k_{bf}\right)\left(k_{nl}-k_{p}\right)-3\phi k_{nl}\left(k_{p}-k_{bf}\right)}\right)k_{bf}.$
Prasher et al. [29]	Aggregation and Brownian, based on Maxwell [14] model, models clumping as a time-dependent process.	$k_{nf}=\left[\frac{k_{a}+2k_{bf}+2\phi_{a}\left(k_{a}-k_{bf}\right)}{k_{a}+2k_{bf}-\phi_{a}\left(k_{a}-k_{bf}\right)}\right]k_{bf},$ $k_{a}={\left(\frac{r_{a}}{r_{p}}\right)}^{d_{f}-3}\left(k_{p}-k_{bf}\right)+k_{bf},$ $\phi_{a}=\frac{\phi }{{\left(r_{a}/r_{p}\right)}^{d_{f}-3}}.$
Xuan et al. [30]	Aggregation and Brownian, considered stationary and dynamic enhancement mechanisms, assumes limited max cluster size.	$k_{nf}=\left(\frac{k_{p}+2k_{bf}+2\phi \left(k_{p}-k_{bf}\right)}{k_{p}+2k_{bf}-\phi \left(k_{p}-k_{bf}\right)}+\frac{\rho_{p}\phi c_{p}}{2k_{bf}}\sqrt{\frac{k_{B}T}{3\pi r_{a}\mu }}\right)k_{bf}.$
Feng et al. [31]	Aggregation and nanolayer, modified Yu and Choi [27] model, considers clusters as spherical 2D lattice.	$k_{nf}=\left(1-\phi_{e}\right)k_{m}+\phi_{e}\left[\left(1-\frac{3}{2}\phi_{e}\right)k_{bf}+\frac{3\phi_{e}k_{bf}}{\alpha }\left(\frac{1}{\alpha }\ln\frac{r_{p}+\delta_{nl}}{\left(r_{p}+\delta_{nl}\right)\left(1-\alpha \right)}-1\right)\right],$ $\phi_{e}=\phi {\left(\beta_{nl}\right)}^{3},$ $\alpha =1-\frac{k_{bf}}{k_{pe}}.$
Wang et al. [32]	Aggregation, assumes a steady distribution of aggregates having varying sizes with 1.5 standard deviation, and geometric mean of particle radius equals the average radius.	$k_{nf}=\left(\frac{1-\phi +3\phi \int_{0}^{\infty }\frac{k_{cl}\left(r_{a}\right)n\left(r_{a}\right)}{k_{cl}\left(r_{a}\right)+2k_{bf}}dr_{a}}{1-\phi +3\phi \int_{0}^{\infty }\frac{k_{bf}n\left(r_{a}\right)}{k_{cl}\left(r_{a}\right)+2k_{bf}}dr_{a}}\right)k_{bf},$ $n\left(r_{a}\right)=\frac{1}{r_{a\sqrt{2\pi }}\ln\left(1.5\right)}\exp\left[-{\left(\frac{\ln\left(r_{a}/r_{p}\right)}{\sqrt{2\pi }\ln\left(1.5\right)}\right)}^{2}\right],$ $k_{cl}\left(r_{a}\right)=\left(3f\left(r_{a}\right)-1\right)k_{p}+\left[3\left(1-f\left(r_{a}\right)\right)-1\right]k_{bf}+\sqrt{\Delta },$ $\Delta ={\left(3f\left(r_{a}\right)-1\right)}^{2}k_{p}^{2}+{\left[3\left(1-f\left(r_{a}\right)\right)-1\right]}^{2}k_{bf}^{2}+2\left[2+9f\left(r_{a}\right)\left(1-f\left(r_{a}\right)\right)\right]k_{p}k_{bf},$ $f\left(r_{a}\right)={\left(\frac{r_{a}}{r_{p}}\right)}^{d_{f}-3}.$
Evans et al. [33]	MDS, assumes stationary, non-aggregating particles, different levels of wetting used in simulations.	$k_{nf}=k_{bf}\left(1+3\phi \frac{\epsilon -1}{\epsilon +2}\right),$ $\epsilon =\frac{r_{p}}{h}.$
Vladkov and Barrat [34]	MDS, considers effects of interfacial resistance, assumes interfacial thermal thickness to be unity.	$k_{nf}=k_{bf}\left(\frac{\left(\frac{k_{p}}{k_{bf}}\left(1+2\Omega \right)+2\right)+2\phi \left(\frac{k_{p}}{k_{bf}}\left(1-\Omega \right)-1\right)}{\left(\frac{k_{p}}{k_{bf}}\left(1+2\Omega \right)+2\right)-\phi \left(\frac{k_{p}}{k_{bf}}\left(1-\Omega \right)-1\right)}\right),$ $\Omega =\frac{R_{k}k_{bf}}{r_{p}}=\frac{1}{r_{p}}.$
Lin et al. [35]	MDS, used Cu–EG simulations, considers both EMT and the nanolayer, assumes spherical particles.	$k_{nf}=k_{bf}+\frac{\left(k_{p}-k_{bf}\right)\left(\frac{\left(1-\zeta_{1}\right)\left(1-\zeta_{2}\right)}{1+2\rho_{nf}\zeta_{1}\zeta_{2}}\right)\phi_{p}+\left(k_{nl}-k_{bf}\right)\left(\frac{1-\zeta_{1}}{1+2\rho_{nf}\zeta_{1}\zeta_{2}}\right)\left(\phi_{pl}-\phi_{p}\right)}{\left(\frac{\left(1-\zeta_{1}\right)\left(1-\zeta_{2}\right)}{1+2\rho_{nf}\zeta_{1}\zeta_{2}}\right)\phi_{p}+\left(\frac{1-\zeta_{1}}{1+2\rho_{nf}\zeta_{1}\zeta_{2}}\right)\left(\phi_{pl}-\phi_{p}\right)+\left(1-\phi_{pl}\right)},$ $\zeta_{1}=\frac{k_{p}-k_{nl}}{k_{p}+2k_{nl}},$ $\zeta_{2}=\frac{k_{nl}-k_{bf}}{k_{p}+2k_{nl}}.$
Mirmohammadi et al. [36]	MDS, simulated Ag–water nanofluid, accounts for particle shape.	$k_{nf}=k_{bf}\left(1+9.89{\left(\frac{T}{300}\right)}^{4.75}{\left(\phi \right)}^{0.65}{\left(0.30855\frac{A_{s}}{V}\right)}^{0.23}\right).$
Chamkha et al. [37]	Hybrid EMT, modified Maxwell [14] model, particle properties replaced with averages.	$k_{hnf}=k_{bf}\left[\frac{\frac{\phi_{1}k_{p,1}+\phi_{2}k_{p,2}}{\phi_{hnf}}+2k_{bf}+2\left(\phi_{1}k_{p,1}+\phi_{2}k_{p,2}\right)-2k_{bf}\phi_{hnf}}{\frac{\phi_{1}k_{p,1}+\phi_{2}k_{p,2}}{\phi_{hnf}}+2k_{bf}+\left(\phi_{1}k_{p,1}+\phi_{2}k_{p,2}\right)-k_{bf}\phi_{hnf}}\right],$ $\phi_{hnf}=\phi_{1}+\phi_{2}.$
Devi and Devi [38]	Hybrid EMT, modified Hamilton and Crosser [15] model, treats base fluid as separate nanofluid.	$k_{hnf}=k_{bf}\left[\frac{k_{p,1}+\left(n_{1}-1\right)k_{bf}-\left(n_{1}-1\right)\phi_{1}\left(k_{bf}-k_{p,1}\right)}{k_{p,1}+\left(n_{1}-1\right)k_{bf}+\phi_{1}\left(k_{bf}-k_{p,1}\right)}\right]\left[\frac{k_{p,2}+\left(n_{2}-1\right)k_{bf}-\left(n_{2}-1\right)\phi_{2}\left(k_{bf}-k_{p,2}\right)}{k_{p,2}+\left(n_{2}-1\right)k_{bf}+\phi_{2}\left(k_{bf}-k_{p,2}\right)}\right].$
Chougule and Sahu [39]	Hybrid Brownian, accounts for particle size, similar technique to Kumar et al. [24]	$k_{hnf}=k_{bf}\left[1+\frac{k_{p,1}\phi_{1}r_{bf}}{k_{bf}\left(1-\phi_{hnf}\right)r_{p,1}}+\frac{k_{p,2}\phi_{2}r_{bf}}{k_{bf}\left(1-\phi_{hnf}\right)r_{p,2}}\right].$
Present	Hybrid Brownian, utilizes work of Patel et al. [23], valid for higher-order nanofluids.	$k_{Nhnf}=k_{bf}\overset{N}{\underset{i=1}{\prod }}\left(1+0.135{\left(\frac{k_{p,i}}{k_{bf}}\right)}^{0.273}\phi_{i}^{0.467}{\left(\frac{T}{20}\right)}^{0.547}{\left(\frac{100}{d_{p,i}}\right)}^{0.234}\right).$

**Table A3:** Summary of nanofluid viscosity models.

**Source**	**Description**	**Model**
Brinkman [48]	Modified Einstein model, assumes spherical particles.	$\mu_{nf}=\frac{\mu_{bf}}{{\left(1-\phi \right)}^{2.5}}.$
Meybodi et al. [49]	Empirical correlation based on data for oxide–water nanofluids.	$\mu_{nf}=\mu_{bf}\times \frac{133.5406-343.8241\exp\left(\phi /d_{p}\right)+290.1180\exp{\left(\phi /d_{p}\right)}^{2}-78.9931\exp{\left(\phi /d_{p}\right)}^{3}}{0.9116+32.3301\left(\frac{\ln\left(d_{p}\right)}{T}\right)-11.7325\left(\frac{\ln{\left(d_{p}\right)}^{2}}{T}\right)}.$
Masoumi et al. [50]	Brownian, assumes a homogeneous distribution of particles and no inter-particle interaction.	$\mu_{nf}=\mu_{bf}+\frac{\rho_{p}u_{B}d_{p}^{2}}{72C\xi },$ $u_{B}=\frac{1}{d_{p}}\sqrt{\frac{18k_{b}T}{\pi \rho_{p}d_{p}}},$ $\xi =\sqrt[{3}]{\frac{\pi }{6\phi }}d_{p}.$
Hosseini et al. [51]	Nanolayer, empirical correlation based on Al_2_O_3_–water nanofluid data.	$\mu_{nf}=\mu_{bf}\left(\exp\left[0.72-0.485\left(\frac{T}{T_{0}}\right)+14.94\phi {\left(\frac{d_{p}+2\delta_{nl}}{d_{p}}\right)}^{3}+0.0105\left(\frac{d_{p}}{1+\delta_{nl}}\right)\right]\right).$
Klazly and Bognár [52]	Brownian and nanolayer, empirical correlation based on wide range of existing data.	$\mu_{nf}=\mu_{bf}\left(\frac{\sqrt{\frac{18k_{b}T\rho_{p}}{\pi }}}{39.6231\sqrt[{3}]{\frac{\pi }{\phi }}C\sqrt{d_{p}}}+1+9.4974\phi_{e}+77.811\phi_{e}^{2}+0.9514\phi_{e}^{3}\right),$ $\phi_{e}=\phi \beta_{nl}^{3},$ $C=\left(2\times 10^{-16}\right)T^{-B_{1}}+\left(3\times 10^{-8}\right)\phi^{-B_{2}}.$
